# Phosphorylation regulates the Star-PAP-PIPKIα interaction and directs specificity toward mRNA targets

**DOI:** 10.1093/nar/gkv676

**Published:** 2015-07-02

**Authors:** Nimmy Mohan, Sudheesh AP, Nimmy Francis, Richard Anderson, Rakesh S. Laishram

**Affiliations:** 1 Cancer Research Program, Rajiv Gandhi Centre for Biotechnology, Thycaud Post, Poojappura, Trivandrum 695014, India; 2 School of Medicine and Public Health, University of Wisconsin-Madison, WI 53706, USA

## Abstract

Star-PAP is a nuclear non-canonical poly(A) polymerase (PAP) that shows specificity toward mRNA targets. Star-PAP activity is stimulated by lipid messenger phosphatidyl inositol 4,5 bisphoshate (PI4,5P_2_) and is regulated by the associated Type I phosphatidylinositol-4-phosphate 5-kinase that synthesizes PI4,5P_2_ as well as protein kinases. These associated kinases act as coactivators of Star-PAP that regulates its activity and specificity toward mRNAs, yet the mechanism of control of these interactions are not defined. We identified a phosphorylated residue (serine 6, S6) on Star-PAP in the zinc finger region, the domain required for PIPKIα interaction. We show that S6 is phosphorylated by CKIα within the nucleus which is required for Star-PAP nuclear retention and interaction with PIPKIα. Unlike the CKIα mediated phosphorylation at the catalytic domain, Star-PAP S6 phosphorylation is insensitive to oxidative stress suggesting a signal mediated regulation of CKIα activity. S6 phosphorylation together with coactivator PIPKIα controlled select subset of Star-PAP target messages by regulating Star-PAP-mRNA association. Our results establish a novel role for phosphorylation in determining Star-PAP target mRNA specificity and regulation of 3′-end processing.

## INTRODUCTION

Eukaryotic messenger RNA (mRNA) maturation involves addition of a poly(A) tail (∼250 adenosine residue) at the 3′-end by poly(A) polymerases (PAPs) in the nucleus (1–5). Almost all pre-mRNAs are polyadenylated by two tightly coupled reactions - cleavage and polyadenylation that involves a large number of protein components. Some of the important factors include cleavage and polyadenylation specificity factor (CPSF), cleavage stimulatory factor (CstF), cleavage factor Im, IIm, symplekin, poly(A) binding protein (PABP) and PAP (5–9). Poly (A) tail at the 3′-end of an mRNA is essential for its stability, translation and export of the transcript from the nucleus to the cytoplasm (2,5,10). Polyadenylation activity of a PAP is regulated by a number of factors including phosphorylation status (2). Phosphorylation of PAPα at the C-terminal domain was reported to inhibit its catalytic activity (11–14). Another nuclear PAP, Star-PAP is also phosphorylated by protein kinases that modulate its activity (2,15). Casein kinase I (CKI) and protein kinase Cδ associate with and phosphorylate Star-PAP (16–18). In addition, there is evidence for other kinases that phosphorylate Star-PAP that are not yet identified (16,17).

Isoforms of CKI α and ϵ phosphorylate Star-PAP in the proline rich region (PRR) in the catalytic PAP domain that is required for the expression of Star-PAP target mRNAs (17). This phosphorylation at the PRR is stimulated by oxidative stress. Both CKIα and ϵ are functionally redundant for this Star-PAP phosphorylation but complement each other to regulate Star-PAP activity (17). CKI isoforms are members of acidic casein kinase I family of serine/threonine kinases (19–21). CKI phosphorylates a large number of protein substrates and the selection of substrate protein is apparently regulated by localization or docking site on the substrate (20,21). All CKI family members recognize a distinct motif for phosphorylation (S/T-P XX S/T, where S/T-P represents phosphoserine or phosphothreonine, and underline S/T represents the CKI target site), indicating that CKI requires a priming phosphorylation (22–25). However, substrate proteins where CKI phosphorylates in the absence of priming phosphorylation have been identified (26–30). In addition to its localization in vesicular structures, synaptic vesicles, centrosome, mitotic spindle, CKIα is present in nuclear speckles and regulates 3′-end processing of Star-PAP target mRNAs, and CKIα is regulated by PI4,5P_2_ (16,31–33). CKIϵ is a ubiquitous Ser/Thr kinase that phosphorylates a variety of proteins including itself (21). CKIϵ autophosphorylates its C-terminus to inhibit the kinase activity (34–36). CKIϵ phosphorylates the circadian protein Period (Per) and the Wnt signaling protein Dishevelled (Dsh) (37–39), and has been implicated in various cellular and nuclear processes (21,40–45).

Star-PAP is a nuclear PAP that is activated by the lipid messenger PI4,5P_2_. Star-PAP regulates specific messages in the cell, a subset of which are those involved in oxidative stress response (46). However, the mechanism of Star-PAP target mRNA specificity is unclear. HO-1 and NQO-1 are two Star-PAP specific mRNAs having roles in oxidative stress response (47–51). HO-1 expression is regulated by CKIα mediated phosphorylation of Star-PAP that is induced by oxidative stress. CKIϵ also phosphorylates Star-PAP and complements with CKIα to regulate oxidative stress stimulated expression of HO-1 mRNA (17). Yet, HO-1 expression is independent of the DNA damage signal induced pathway that regulates Star-PAP through the serine kinase PKCδ controlling the 3′-end processing of apoptotic gene BIK. This indicates a signal specific regulation of target mRNA expression by the association of specific kinase coactivators (18).

Star-PAP polyadenylation activity is stimulated by both oxidative stress and PI4,5P_2_ (46). Apart from its polyadenylation activity, Star-PAP can add short U-tail to the U6 snRNA involved in splicing (52). Star-PAP shares similar domain architecture with non-canonical PAPs but functions most like canonical PAPs (46,53). It has a PAP (catalytic) domain split by a PRR (∼200 amino acids) where several serine/threonine sites are putative targets for CKIα/ϵ followed by a PAP associated domain (2). Star-PAP has two RNA binding motifs at the N-terminus, zinc finger (ZF) and RNA recognition motif (RRM) both of which are involved in RNA binding (53). Star-PAP complex also contains unique components such as PIPKIα and kinases including CKIα/ϵ and PKCδ (16–18,46). PIPKIα localizes with Star-PAP in the nuclear speckle and together regulate select mRNA expression (46). Yet, microarray data indicated that only a set of messages were common between Star-PAP and PIPKIα knockdown, and a large number of Star-PAP target transcripts were insensitive to PIPKIα knockdown and *vice versa* (46).

In this study, we report an *in vivo* Star-PAP phosphorylated residue (serine 6) in the ZF region at the N-terminus. The phospho-deficient (serine 6 to alanine, S6A) but not phosphomimetic (Serine 6 to glutamate, S6E) mutation of Star-PAP resulted in reduced expression and 3′-end formation of target HO-1 or NQO-1 mRNA. S6A mutation also inhibited Star-PAP mRNA binding and interaction with PIPKIα. The S6 phosphorylation was characterized and we show that CKIα phosphorylates the S6 residue on Star-PAP *in vivo*. S6-phosphorylated Star-PAP is largely nuclear speckle localized and this phosphorylation regulates Star-PAP RNA association with specific pre-mRNAs (HO-1 and NQO-1). Unlike other CKIα phosphorylation site(s) at the PRR on Star-PAP, S6 phosphorylation is independent of oxidative stress signal. S6-phosphorylated Star-PAP shares target mRNAs with those regulated by PIPKIα. We demonstrate that S6 phosphorylation regulates the Star-PAP PIPKIα interaction that in turn is required for specific mRNA expression. Our results demonstrate a mechanism where Star-PAP target mRNA specificity is mediated through specific phosphorylation that controls association with the coactivator PIPKIα.

## MATERIALS AND METHODS

### Cell culture, transfections and cell stimulation

Human embryonic kidney 293 and HeLa cell lines were obtained from American Type Culture Collection and maintained in Dulbecco's Modified Eagle's Medium with 10% fetal bovine serum (FBS) and penicillin/streptomycin (50 u/ml) at 37°C in 5% CO_2_. Cells were transfected using Oligofectamine (Invitrogen) for siRNA knockdown and Lipofectamine (Invitrogen) for plasmid DNA as per manufacturer's instructions. The knockdown of Star-PAP, CKIα or PIPKIα was carried out as described previously (16,46). siRNA oligos used are listed in Supplementary Data. To induce a transcriptional antioxidant response, cells were treated for 4 h with 100 μM *tert*-butylhydroquinone (tBHQ) in dimethyl sulfoxide (DMSO). Control cells were treated with solvent DMSO to a final concentration 0.1%. The CKIα activity inhibitors CKI7 (IC_50_ ∼ 6 μM) and IC261 (IC_50_ ∼ 11 μM) were dissolved in DMSO and used at a concentration of 100 μM each to treat the cells for 3 h to inhibit the CKI activity in the cell. Other kinase inhibitors were used at concentrations as indicated.

### Immunoprecipitation and immunoblot analysis

Total cell lysates were prepared in buffer A (50 μM Tris [pH 7.4], 100 mM KCl, 5 mM ethylenediaminetetraacetic acid (EDTA), 5% NP-40, 5 mM NaF, 100 μg/ml RNase A, 1 mM NaVO_4_, 50 mM β glycerol phosphate and protease inhibitor) and gently sonicated. Immunoprecipitations from the lysate and immublottings were carried out as described previously (18). For immunoprecipitation (IP) from cytoplasmic and nuclear extracts, nuclear fractionation was carried out using NE-PER nuclear and cytoplasmic fractionation kit (Pierce, Thermo Scientific). The images of the western blot were quantified using image J software. Input = 10% of the total protein used for IP. List of antibodies used are shown in Supplementary Data.

### Expression and affinity purification of Star-PAP

Affinity purified human FLAG-Star-PAP was obtained from HEK-293 cells with stably expressed FLAG-Star-PAP or S6A mutant FLAG-Star-PAP by anti-FLAG affinity purification system using anti-FLAG (M2) agarose (Sigma) and 3X FLAG as described previously (46). To identify the phosphorylation sites, the purified FLAG-Star-PAP was phosphoenriched, followed by mass spectrometry sequencing at the UW-Madison mass spectrometry facility (http://www.biotech.wisc.edu/services/massspec). His- and Glutathione S-Transferase (GST)- tagged PIPKIα, Star-PAP or various Star-PAP deletions were obtained by overexpression in *Escherichia coli BL21*(*DE3)* and *BL21* respectively after induction with 0.5 mM IPTG at 18°C overnight as described previously (53). Purified proteins were concentrated with PEG, snap frozen and stored in −80°C.

### GST pulldown assay

GST fusions with full length (FL) Star-PAP, ZF domain, RRM domain, ZF-RRM domains of Star-PAP or GST-PIPKIα and control GST proteins were immobilized on glutathione-Sepharose beads by overnight incubation of overexpressed *E. coli (BL21)* lysates with glutathione-sepharose beads (Invitrogen) pre-equilibriated in TEN 100 buffer (20 mM Tris, pH 7.5, 0.1 mM EDTA and 100 mM NaCl). The GST-tagged protein bound beads were washed and incubated with the *E. coli* extracts with overexpressed His-PIPKIα or Star-PAP deletions (FL, ZF, RRM or ZF-RRM domains) as described previously (53). Buffers were supplemented with protease inhibitor cocktail (Roche), DNaseI and RNaseA to rule out interactions through nucleic acids. The inputs show 10% of the lysates used for the pull down.

### 3′-RACE assay and 3′-end cleavage measurement

Total cellular RNA was isolated using the RNAeasy mini Kit (Qiagen) and cDNA for 3′-RACE assays was synthesized using the 3′-RACE system (Invitrogen) according to manufacturer's instructions with 2 μg of total RNA. The products were resolved on agarose gel, cloned and confirmed by sequencing. For measurement of cleavage efficacy, uncleaved mRNA levels were measured by quantitative real time polymerase chain reaction (PCR) using a pair of primers across the cleavage site as described earlier (46). The non-cleaved messages were expressed as fold change over the total mRNA. The primers used for 3′-RACE and cleavage assays are shown in Supplementary Data.

### RNA immunoprecipitation (RIP)

RNA immunoprecipitation (RIP) experiments were performed as described previously (53). The specific primer used to assess the association of FLAG-Star-PAP, RNA Pol II or mutant Star-PAP is shown in Supplementary Data. Quantitative RIP experiments were carried out using quantitative real-time PCR (qRT-PCR) as described earlier (54,55).

### Quantitative real-time PCR (qRT-PCR)

Total cellular RNA was extracted, reverse transcribed and target mRNA was quantified with the CFX98 multi-color system using SYBR Green Supermix (Bio-Rad) as described previously (18). Single-product amplification was confirmed by melting-curve analysis and primer efficiency was near 100% in all experiments. Quantification is expressed in arbitrary units and target mRNA abundance was normalized to the expression of GAPDH with the Pfaffl method. All qRT-PCR results were representative of at least three independent experiments. Primers used for qRT-PCRs are indicated in Supplementary Data.

### Immunofluorescence microscopy

HeLa cells were used for immunofluorescence (IF) experiments with required knockdowns or transfections as indicated in the text. Samples for the IF were prepared as described earlier (46) and images were taken on Nikon A1R laser scanning confocal microscope. Antibodies used are shown in Supplementary Data.

### RNA EMSA experiment

Uniformly radiolabeled NQO-1 or HO-1 UTR RNA substrates were prepared by *in vitro* transcription of the linearized template plasmid pTZ-HO-1 (53) extending from ∼150 nt upstream and ∼140 nt downstream of poly(A) site under T7 promoter or pTZ-NQO-1 (having equivalent NQO-1 UTR region) using T7 transcription kit (Fermentas). For electrophoretic mobility shift assay (EMSA) using FLAG-affinity purified Star-PAP, we used only the Star-PAP binding region (footprint) upstream of poly(A) signal from HO-1 or equivalent UTR RNA from CHAC1 UTR as template. EMSA experiments were carried out as described earlier (53) each in 20 μl EMSA-binding buffer containing 0.5 nM of uniformly labeled RNA and Star-PAP in presence of increasing PI4,5P_2_ concentrations as indicated.

### *In vitro* kinase assay

*In vitro* kinase assays were performed as described previously (17). Casein Kinase IIα (NEB) 250 units or affinity purified FLAG-CKIα (10 μM) were used in each *in vitro* kinase reaction. FLAG-Star-PAP (5 μM) each was heat inactivated at 65°C for 20 min before using as substrate in each kinase assay reaction. Four microgram each of Star-PAP N-terminal peptide and casein were used for kinase reaction with CKIα and CKIIα. All reactions were analyzed on 10% Sodium dodecyl sulphate-polyacrylamide gel electrophoresis (SDS-PAGE) except the short peptide which was analyzed on 25% SDS-PAGE gel.

## RESULTS

### Phosphorylation of Star-PAP in the ZF-region and its role in mRNA 3′-end processing

**Figure 1.**
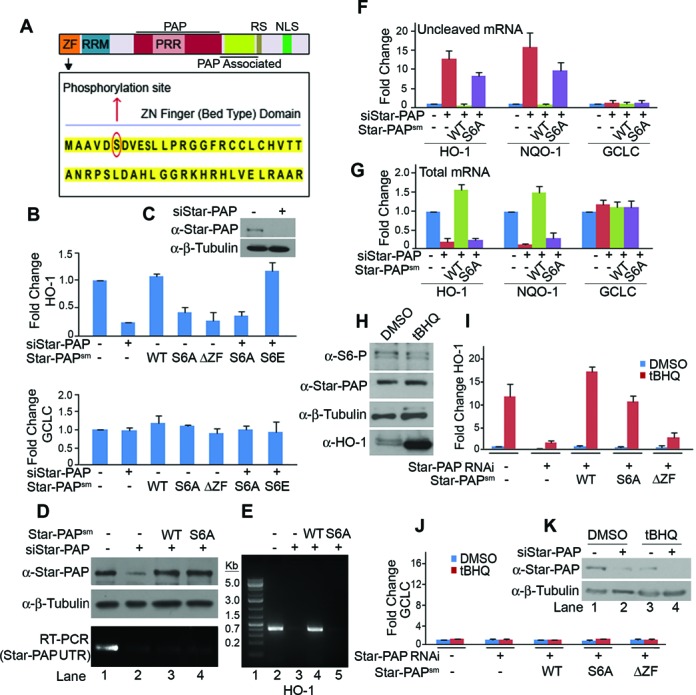
Phosphorylation of Star-PAP at Ser-6 controls expression of Star-PAP target mRNAs and is insensitive to oxidative stress stimulation. (**A**) Schematic of Star-PAP domain architecture showing the ZF sequence and S6 phosphorylation site. (**B**) qRT-PCR of HO-1 (upper) and GCLC (lower) with total RNA isolated from HEK 293 cells with transiently expressed exogenous wild-type (WT), S6A (serine 6 to alanine mutation), S6E (serine 6 to glutamate) or ΔZF (ZF, 1–50 amino acid deletion) Star-PAP in presence (+) or absence (−) of Star-PAP knockdown as indicated. (**C**) Western blot showing knockdown of Star-PAP. (**D**) Western blot showing relative amount of endogenous Star-PAP and exogenously expressed Star-PAP^WT^ or Star-PAP^S6A^ using Star-PAP antibody or internal control β-Tubulin from HEK 293 cells after Star-PAP knockdown as indicated. Quantification is shown in Supplementary Figure S2A and corresponding qRT-PCR in Supplementary Figure S2B. Bottom gel indicates RT-PCR using specific primers at the 3′-UTR which is absent in the transfected Star-PAP^sm^ constructs. (**E**) 3′-RACE assay of HO-1 from total RNA isolated after Star-PAP knockdown followed by rescue with various Star-PAP constructs in stably expressed HEK 293 cells as indicated. (**F**) Uncleaved pre-mRNA levels of HO-1, NQO-1 and GCLC expressed relative to the total mRNA and (**G**) total mRNA levels after Star-PAP knockdown in presence of stably expressed Star-PAP constructs as indicated. (**H**) Western blot analysis on lysates from HEK 293 cells after treatment with tBHQ or solvent control DMSO with antibodies as indicated. (**I**) qRT-PCR analysis of HO-1 and (**J**) GCLC mRNA from HEK 293 cells after Star-PAP knockdown in presence of stably expressed Star-PAP constructs as indicated. (**K**) Western blot indicating knockdown of Star-PAP in presence and absence of tBHQ treatment. Wherever (−) siRNA is indicated in the figure, we have used control scrambled siRNA.

Previously, CKIα/ϵ mediated phosphorylation of Star-PAP in the PRR within the catalytic domain was reported to control expression of Star-PAP target mRNAs (16,17). To identify the *in vivo* phosphorylation sites on Star-PAP, we purified Star-PAP from stably expressed FLAG-tagged Star-PAP from HEK 293 cells by anti-FLAG affinity purification (46) and subjected to mass spectrometry analysis (Supplementary Figure S1A). Cells were either treated with oxidative stress agonist *tert*-Butylhydroquinone, tBHQ to induce oxidative stress response or solvent control DMSO before isolation of FLAG Star-PAP. We identified a phosphorylated serine, the sixth amino acid (S6) from the N-terminus at the ZF-region under both normal and oxidative stress stimulated conditions. Domain architecture of Star-PAP and sequence of the ZF-region with S6 phosphorylation site is shown in Figure 1A and Supplementary Figure S1B. Also, the predicted structure of the ZF-motif (1–50 amino acids) obtained using ITASSER protein structure prediction program is shown in Supplementary Figure S1B and the position of S6 site is indicated in red.

To study the functional significance of S6 phosphorylation, we mutated the serine 6 to alanine (S6A) (Star-PAP^S6A^) or glutamate (S6E) (Star-PAP^S6E^) on FLAG-Star-PAP having silent mutations (sm) that will render it insensitive to siRNA oligos used for Star-PAP knockdown (18). We analyzed the expression profile of Star-PAP *bona fide* targets HO-1 and NQO-1 mRNA. As expected, knockdown of Star-PAP (Figure 1C) resulted in a loss of HO-1 mRNA level (Figure 1B). Transient expressions of Star-PAP^S6A^ or control ZF deletion (Star-PAP^ΔZF^) caused a similar loss (∼60–70% reduction) of HO-1 mRNA level (Figure 1B) indicating that S6 phosphorylation is required for Star-PAP basal target mRNA expression. Moreover, expression of Star-PAP^S6A^ in HEK 293 cells after knockdown of Star-PAP also resulted in a similar diminished HO-1 mRNA level (Figure 1B). There was slightly higher expressions of transfected Star-PAP^S6A^ or Star-PAP^WT^ than the endogenous Star-PAP level (Figure 1D, Supplementary Figure S2A and B). Interestingly, expression of phosphomimetic mutation (Star-PAP^S6E^) rescued the loss of HO-1 expression after Star-PAP knockdown (Figure 1B). There was no effect of Star-PAP knockdown or expressions of Star-PAP^S6A^ on mRNA levels of non-target GCLC (Figure 1B, lower panel).

Stable expression of wild-type FLAG-Star-PAP (Star-PAP^WT^) but not Star-PAP^S6A^ rescued the loss of HO-1 mRNA level after Star-PAP knockdown (Supplementary Figure S1E). Similarly, Star-PAP^WT^ but not Star-PAP^S6A^ rescued the 3′-end formation in a 3′-RACE assay of two Star-PAP target messages HO-1 (Figure 1E) and NQO-1 (Supplementary Figure S1C). The rapid amplification of cDNA ends (RACE) products of non-target GAPDH did not show any difference either in presence of Star-PAP^WT^ or Star-PAP^S6A^ (Supplementary Figure S1D). We then analyzed the cleavage efficiency of HO-1 and NQO-1 by measuring the uncleaved pre-mRNA level using a pair of primers across the cleavage site in a similar rescue experiment with WT or S6A mutant Star-PAP. Consistent with our earlier findings, knockdown of Star-PAP resulted in an increased accumulation of uncleaved HO-1 or NQO-1 pre-mRNA while there was diminished total mRNA level (Figure 1F and G). A similar accumulation was visible in the case of stably expressed Star-PAP^S6A^ but not with Star-PAP^WT^ (Figure 1F) in presence of Star-PAP knockdown confirming that S6 phosphorylation is critical for the 3′-end processing of Star-PAP target pre-mRNAs. Analysis of splicing efficiency of HO-1 mRNA using a pair of primers across the exons of HO-1 or non-target GCLC mRNA showed no effect of Star-PAP knockdown or expression of Star-PAP^S6A^ on HO-1 or GCLC splicing while there was increased accumulation of uncleaved pre-mRNA and loss of total mRNA levels of HO-1 (Supplementary Figure S2G–J). This was further confirmed using a splicing independent reporter construct (devoid of introns) FLAG-NQO-1 expressed from pCMV promoter and driven by specific NQO-1 UTR or control SV40 UTR (Supplementary Figure S2C–F). Moreover, there was no visible effect of Star-PAP S6A mutation on cell viability (52) (data not shown). These results demonstrate that the effect of S6A mutation on HO-1 and NQO-1 is specifically due to Star-PAP mediated regulation of 3′-end processing and not through general splicing.

To further characterize the S6 phosphorylation, an antibody was raised against a S6-phosphorylated Star-PAP N-terminal peptide (MAAVDS(P)DVESLPRG). The purified antibody detected the phosphorylated Star-PAP (referred to as S6-phospho Star-PAP and the antibody as S6-phospho antibody) in HEK 293 cells which was lost upon Star-PAP knockdown similar to Star-PAP antibody (Supplementary Figure S3A). The comparison of the S6-phospho antibody with that of earlier reported Star-PAP antibody is shown in Supplementary Figure S3. Competition with corresponding peptides demonstrated the specificity of the S6-phospho antibody (Supplementary Figure S3B and C). The S6-phospho peptide specifically competed with the S6-phospho antibody but not with our FL or non-phospho N-terminal Star-PAP antibody (N-Term) (Supplementary Figure S3B and C). We also tested the S6-phospho Star-PAP antibody in IF experiments where we detected the S6-phospho Star-PAP along with nuclear speckle marker SC 35 (Supplementary Figure S3D) similar to our earlier established Star-PAP N-Term antibody (46). Knockdown of Star-PAP resulted in the loss of both S6-phospho and total Star-PAP in HeLa cells (Supplementary Figure S3D). Peptide competition also confirmed the specificity of S6-phospho antibody in IF experiments (Supplementary Figure S3E). Moreover, our S6-phospho antibody was further tested in an experiment using transient expressions of FLAG-Star-PAP in HeLa cells. Colabeling of cells with S6-phospho antibody along with anti-FLAG antibody in FLAG-Star-PAP expressing cells showed that S6-phospho protein colocalized with FLAG positive punctae in the nucleus (Supplementary Figure S5C; also Figure 5F) confirming the authenticity and specificity of the S6-phospho antibody.

**Figure 2.**
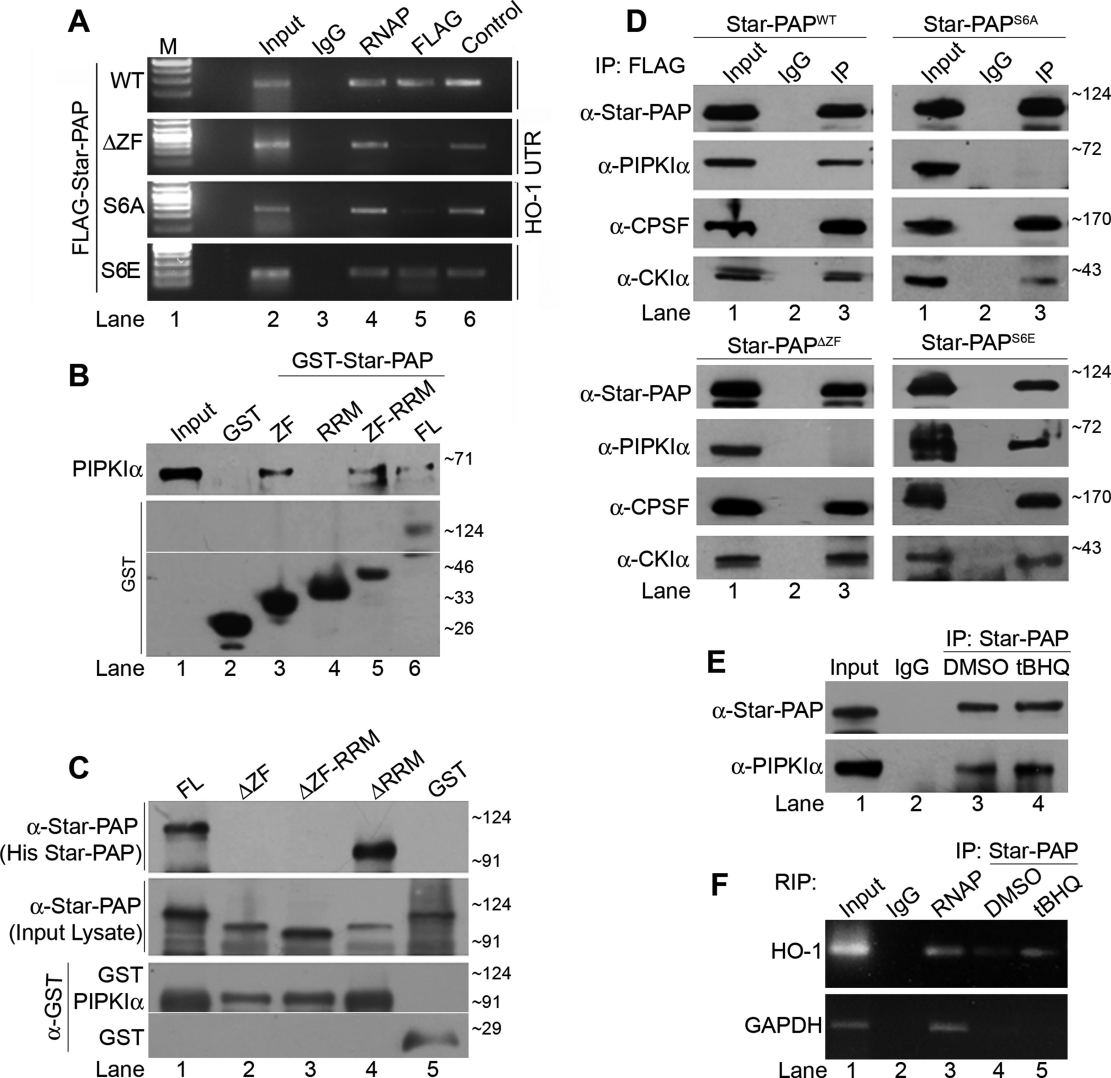
PIPKIα interacts with Star-PAP through ZF-region and Star-PAP S6 phosphorylation regulates both PIPKIα interaction and target mRNA association in the cell. (**A**) RIP analysis of HO-1 using anti- FLAG, -RNA Pol II and -Star-PAP antibodies from HEK 293 cells with exogenously expressed WT, S6A, S6E or ΔZF Star-PAP. Control indicates RIP using Star-PAP antibody from HEK 293 cells. (**B**) GST-pulldown experiments using GST -Star-PAP (FL), -ZF, -RRM or -ZF-RRM domain (indicated on the top) with overexpressed PIPKIα (indicated on the left); and (**C**) GST-pulldown experiments using GST-PIPKIα with overexpressed Star-PAP deletions (ZF, RRM or ZF-RRM) or full length Star-PAP (FL). (**D**) Immunoprecipitation using anti-FLAG antibody from HEK 293 cells with exogenously expressed FLAG -Star-PAP^WT^, -Star-PAP^S6A^, -Star-PAP^ΔZF^ and Star-PAP^S6E^ and analyzed for associated proteins as indicated. (**E**) IP and (**F**) RIP experiment using anti -FLAG antibody in HEK 293 cells with exogenously expressed FLAG-Star-PAP in the presence of tBHQ or DMSO treatment of the cell.

**Figure 3.**
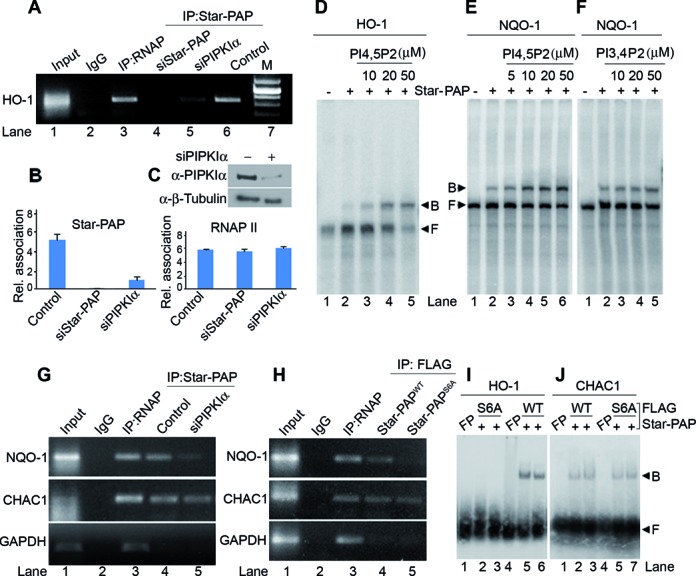
PIPKIα and PI4,5P_2_ modulates Star-PAP RNA binding; and PIPKIα along with S6 Star-PAP phosphorylation regulates selective mRNA association in the cell (**A**) RIP analysis Star-PAP on HO-1 mRNA under conditions as indicated. (**B**) Quantitative RIP of Star-PAP and RNA Pol II on HO-1 under conditions as indicated. (**C**) Western blot showing PIPKIα knockdown is also indicated. (**D**–**F**) EMSA experiment using a an *in vitro* transcribed UTR RNA fragment from HO-1 UTR (D) and equivalent UTR RNA fragment from NQO-1 (E and F) with Star-PAP in presence of (E) increasing concentrations of PI4,5P_2_ (0–50 μM) and (F) PI bis-phosphate control PI3,4P_2_ (0–50 μM) as indicated. (**G** and **H**) RIP analysis as in (A) on mRNAs under the conditions as indicated. (**I** and **J**) EMSA experiment using Star-PAP binding RNA region upstream of poly(A) signal from HO-1 UTR (I) and an equivalent corresponding RNA from CHAC1 UTR (J) with FLAG affinity purified Star-PAP (WT and S6A) from stable HEK 293 cells expressing Star-PAP^WT^ or Star-PAP^S6A^.

**Figure 4.**
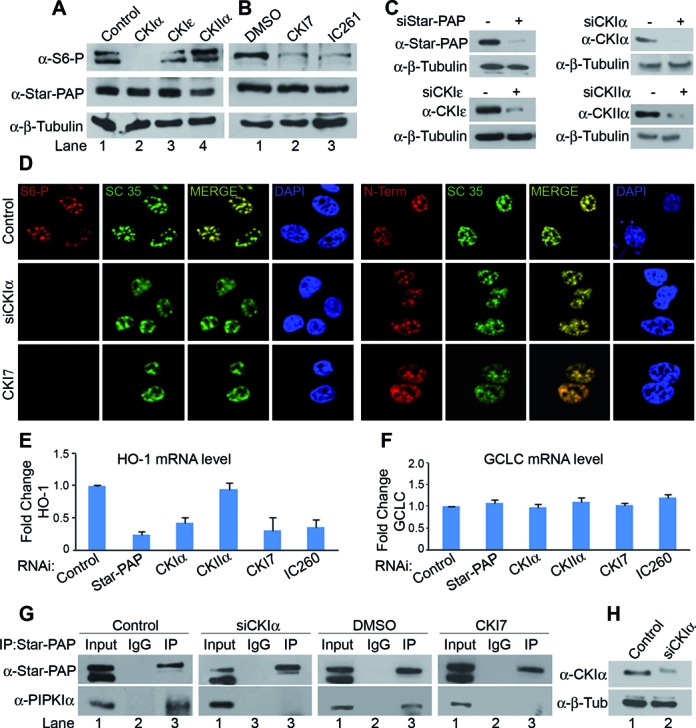
CKIα phosphorylates the S6 residue at Star-PAP ZF-region. (**A**) Western blot analysis of S6-phospho (S6-P) and normal Star-PAP in HEK 293 cells after knockdown of CKIα, CKIϵ or CKIIα or (**B**) treatment with CKI inhibitors CKI7 or IC261. (**C**) Western blot showing knockdown of Star-PAP, CKI isoforms and CKII. Wherever siRNA (−) is indicated, we have used control scrambled siRNA. (**D**) Immunofluorescence analysis of S6-P and normal Star-PAP (N-term) localization compared to nuclear speckle marker SC 35 and DAPI in HeLa cells in the presence of control and CKIα knockdown or CKI inhibitor CKI7. (**E**) Expression profile of HO-1 and (**F**) GCLC mRNA in HEK 293 cells under conditions as indicated (**G**) Analysis of PIPKIα association with Star-PAP after knockdown of CKIα or treatment with CKI7 by immunoprecipitation as in Figure 2D under conditions as indicated. (**H**) Western blot showing knockdown of CKIα.

**Figure 5.**
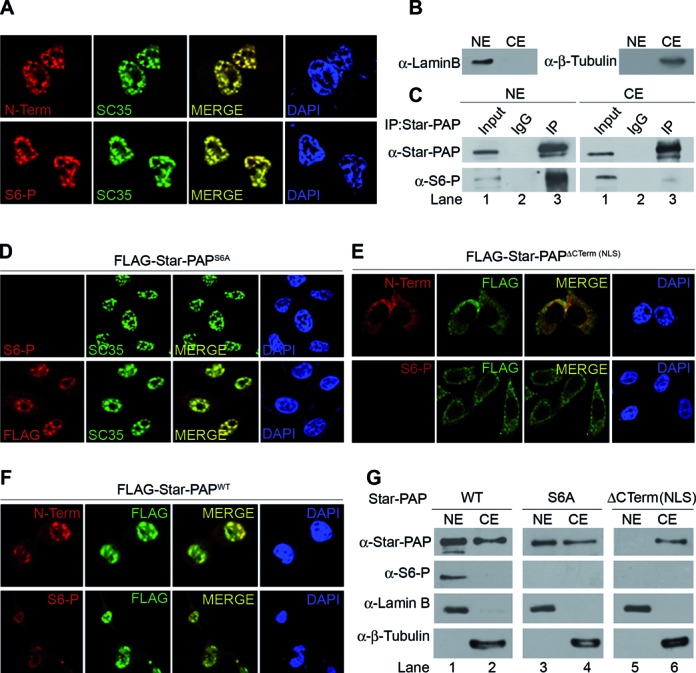
S6 phosphorylation occurs within the nucleus and S6-phospho Star-PAP is retained inside the nucleus. (**A**) IF experiment to probe localization of Star-PAP or S6-phospho Star-PAP as indicated. (**B**) Nuclear fractionation followed by (**C**) IP of Star-PAP from nuclear (NE) and cytoplasmic (CE) extracts. (**D**) IF probing S6 phospho- and FLAG- Star-PAP in HeLa cells transfected with FLAG-Star-PAP S6A mutant, (**E**) -C-term deletion (NLS) Star-PAP or (**F**) -wild-type Star-PAP after knockdown of endogenous Star-PAP in the cell. (**G**) Western analysis of S6-phospho and normal Star-PAP in the nuclear and cytoplasmic extracts of HEK 293 cells in presence of FLAG-Star-PAP (WT, S6A mutant or C-term deletion).

### ZF-phosphorylation is independent of oxidative stress signal stimulated pathway of Star-PAP regulation

S6 phosphorylation was observed under both normal and oxidative stress stimulated conditions in mass spectrometry sequencing. Earlier studies reported stimulation of CKI mediated Star-PAP phosphorylation in the catalytic domain by oxidative stress agonist, tBHQ (17). Therefore, to further test the effect of oxidative stress on Star-PAP S6 phosphorylation, we treated HEK 293 cells with tBHQ or solvent control DMSO and analyzed for S6-phospho Star-PAP by western blot. There was no change in the level of S6-phospho Star-PAP on tBHQ treatment (Figure 1H) suggesting that S6 phosphorylation is independent of oxidative stress signaling. Control HO-1 protein levels were stimulated several fold on tBHQ treatment (Figure 1H). IF experiments also demonstrated no effect of tBHQ treatment on the nuclear localization of S6-phospho Star-PAP (Supplementary Figure S4A).

Further, we examined the levels of HO-1 mRNA in the presence or absence of tBHQ treatment of cells using knockdown-rescue approach. We observed >10-fold induction of HO-1 mRNA expression on tBHQ treatment compared to the control DMSO (Figure 1I). Knockdown of Star-PAP (Figure 1K) resulted in the loss of both stimulated and unstimulated HO-1 expression which was rescued by stable transfection of Star-PAP^WT^ (Figure 1I). Star-PAP^S6A^ also significantly rescued the oxidative stress induced expression of HO-1 mRNA similar to Star-PAP^WT^ (Figure 1I) suggesting that S6-phosphorylation is not involved in the regulation of oxidative stress stimulated HO-1 expression. Nevertheless, there was still a significant loss of basal HO-1 mRNA expression in presence of Star-PAP^S6A^ (Figure 1B and I). Control non-target GCLC expression was not altered by either knockdown or in presence of Star-PAP^S6A^ (Figure 1J). These results illustrate that S6 phosphorylation is independent of oxidative stress induced pathway of Star-PAP regulation.

### Serine 6 phosphorylation in the Star-PAP ZF modulates PIPKIα interaction and Star-PAP RNA binding

Star-PAP ZF-region is involved in Star-PAP RNA binding and the deletion of ZF domain was shown to reduce Star-PAP RNA interaction (53). To further characterize the functional significance of S6 phosphorylation, we examined the association of Star-PAP on target HO-1 UTR RNA by RIP experiment in HEK 293 cells that expressed either FLAG-Star-PAP^S6A^ or -Star-PAP^WT^ using anti-FLAG antibody (53). The associated RNA was then identified with RT-PCR using HO-1 specific primers at the 3′-UTR. As reported earlier (46,53), FLAG-Star-PAP associated with HO-1 UTR RNA (Figure 2A). Phospho-deficient (S6A) but not phosphomimetic (S6E) mutation of Star-PAP resulted in a loss of association of Star-PAP with HO-1 UTR RNA (Figure 2A, Supplementary Figure S4B). The ZF deletion also exhibited a similar reduction in Star-PAP association with HO-1 RNA (Figure 2A) suggesting that S6 phosphorylation is critical for Star-PAP interaction with target pre-mRNA in the cell.

PIPKIα is a coactivator of Star-PAP and generates PI4,5P_2_ that regulates Star-PAP activity (46). We demonstrated the direct interaction of PIPKIα with Star-PAP through ZF-region using GST pulldown experiments (Figure 2B and C). GST-ZF or -ZF-RRM but not -RRM domain of Star-PAP exhibited direct binding to His-PIPKIα similar to the FL Star-PAP (Figure 2B). The ZF-domain on His-Star-PAP was deleted and pulldown experiments were carried out using GST-PIPKIα. FL and RRM domain deletion Star-PAP were efficiently pulled down by GST-PIPKIα (Figure 2C). However, ZF deletion or ZF-RRM deletion abolished association of Star-PAP with GST-PIPKIα (Figure 2C) illustrating that PIPKIα binds Star-PAP at the ZF-region.

Since Star-PAP ZF region is required for PIPKIα interaction, we assayed if S6 phosphorylation regulates PIPKIα binding *in vivo*. We transiently expressed FLAG-Star-PAP^S6A^ or -Star-PAP^WT^ and analyzed the association of Star-PAP with PIPKIα by immunoprecipitation experiment using anti-FLAG antibody. We detected PIPKIα in the immunoprecipitates of WT Star-PAP, however, S6A mutation abolished the association of PIPKIα with Star-PAP similar to the control ZF deletion (Figure 2D). Star-PAP with S6 phosphomimetic mutation (Star-PAP^S6E^), however, showed similar associations with PIPKIα as that of Star-PAP^WT^. Control cysteine mutations in the core ZF structure to serine (C18S, C21S) (see Supplementary Figure S5A) exhibited no effect on Star-PAP PIPKIα interaction (Supplementary Figure S4D) indicating that it is not the general disruption of ZF structure but S6 phosphorylation that regulate PIPKIα binding. There was no effect on the association of other Star-PAP binding partners such as CPSF 160 or CKIα by S6A or S6E mutations (Figure 2D). Moreover, control PRR deletion did not affect the PIPKIα interaction (Supplementary Figure S4C) confirming that S6 phosphorylation is required for Star-PAP-PIPKIα interaction in the cell. It was further confirmed using stable transfections of FLAG-Star-PAP^S6A^ where we showed similar loss of PIPKIα interaction (Supplementary Figure S4E and F). Together these results indicate that Star-PAP S6 phosphorylation modulates both its RNA binding properties and PIPKIα association and expression of target mRNAs in the cell. Moreover, deletion of ZF or S6A mutation of Star-PAP resulted in reduced expression of HO-1 mRNA (Figure 1B) reiterating that Star-PAP-PIPKIα interaction is required for expression of target messages. Further, there was no effect of tBHQ treatment on Star-PAP PIPKIα association (Figure 2E) or Star-PAP association with target HO-1 pre-mRNA (Figure 2F) consistent with our observation that S6-phosphorylation is independent of oxidative stress stimulated pathway of Star-PAP regulation.

### PIPKIα and the lipid messenger PI4,5P_2_ modulates Star-PAP mRNA binding

Although S6 phosphorylation site lies in the ZF region on Star-PAP, it is outside the core ZF motif involved in RNA binding (Supplementary Figures S5A and 1A). Yet, S6 phosphorylation appears to alter Star-PAP RNA interaction (Figure 2A). Therefore, we explored the indirect effect of S6 phosphorylation on target mRNA association through PIPKIα interaction with Star-PAP. To assess this, the effect of PIPKIα interaction or its product lipid messenger PI4,5P_2_ on Star-PAP mRNA association was measured. PIPKIα was knocked down in HEK 293 cells (Figure 3C) and studied Star-PAP binding with HO-1 UTR RNA in the cell by RIP experiment. As shown in Figure 3A, we observed an association of Star-PAP with HO-1 RNA similar to that of RNA Pol II, yet, PIPKIα knockdown resulted in a significant reduction in Star-PAP HO-1 mRNA association compared to the control cells. Quantitatitive RIP analysis showed ∼70% reduction of Star-PAP association with HO-1 RNA on PIPKIα knockdown, while there was no difference in control RNA Pol II association with the HO-1 RNA (Figure 3B) indicating that PIPKIα regulates Star-PAP RNA binding in the cell. The role of the lipid messenger PI4,5P_2_ (generated by PIPKIα in the nucleus) was assayed in an RNA EMSA experiment with His-Star-PAP and an *in vitro* transcribed HO-1 or NQO-1 UTR RNA in presence of increasing PI4,5P_2_ concentration (5 to 50 μM) or control bisphospho-inositide PI3,4P_2_. Consistent with earlier findings, we observed direct Star-PAP binding to HO-1 (53) and also NQO-1 UTR RNA (Figure 3D–F). Intriguingly, there was increase in the binding efficiency/affinity of Star-PAP to both HO-1 and NQO-1 UTR RNA on increasing PI4,5P_2_ addition (Figure 3D and E) with no effect on PI3,4P_2_ addition (Figure 3F) indicating that PI4,5P_2_ modulates Star-PAP RNA binding.

### Star-PAP PIPKIα interaction and S6 phosphorylation determines specificity of Star-PAP-target mRNA association

Since PIPKIα and S6 phosphorylation are involved in Star-PAP mRNA association, we tested Star-PAP association with earlier reported PIPKIα insensitive Star-PAP target mRNA, CHAC1 (16). RIP analysis was performed in HEK 293 cells with stably expressed FLAG-Star-PAP^S6A^ or -Star-PAP^WT^ and association of Star-PAP was analyzed using targets (HO-1, NQO-1 and CHAC1). While there was normal association with Star-PAP^WT^ to all the mRNAs studied (HO-1, NQO-1, CHAC1), Star-PAP^S6A^ exhibited diminished association with NQO-1 (Figure 3H) and HO-1 UTR RNA (Figure 2A). However, there was no effect of the S6A mutation on Star-PAP association with CHAC1 UTR RNA (Figure 3H). EMSA experiment with FLAG affinity purified Star-PAP also demonstrated association with Star-PAP binding region from HO-1 UTR or an equivalent UTR RNA from CHAC1 (Figure 3I, J). There was considerable reduction in the binding on HO-1 (Figure 3I) by S6A mutation with no effect of the mutation on CHAC1 RNA (Figure 3J). Remarkably, RIP analysis after knockdown of PIPKIα also resulted in diminished association of Star-PAP with HO-1 (Figure 3A) and NQO-1 mRNA (Figure 3G). However, Star-PAP-CHAC1 association was independent of PIPKIα knockdown (Figure 3G) similar to S6A Star-PAP mutation indicating that both S6 phosphorylation and PIPKIα together regulates specificity of Star-PAP target mRNA association. Star-PAP either WT or with S6A mutation, or in the presence of PIPKIα knockdown in the cell did not show any association with non-target GAPDH (Figure 3G and H).

### CKIα phosphorylates serine 6 on Star-PAP ZF to regulate Star-PAP target mRNAs

Sequence analysis reveals that S6-phosphorylation region (SDVES) on Star-PAP is a putative site for a negative charge directed kinase such as casein kinase. We previously showed that CKI isoforms α and ϵ are present in the complex and phosphorylates Star-PAP in the catalytic domain, however, the exact phosphorylation residues were unknown (17). Therefore, to identify the kinase that phosphorylates S6, the serine kinases—CKI isoforms α and ϵ, and casein kinase IIα were knocked down in HEK 293 cells (Figure 4C), and then looked for phosphorylation of Star-PAP with S6-phospho antibody by western blot analysis. Knockdown of CKIα but not CKIϵ or CKIIα resulted in the loss of S6-phospho Star-PAP (Figure 4A). There was no effect of the knockdown on the levels of S6 unphosphorylated Star-PAP (we now refer to it as normal Star-PAP thereafter in this paper) (Figure 4A) indicating that CKIα is the kinase that phosphorylates S6 in the Star-PAP ZF region. It was further confirmed using CKI specific inhibitors—CKI7 and IC261, both of which resulted in diminished S6-phospho Star-PAP but did not affect the normal Star-PAP levels (Figure 4B). Furthermore, we investigated localization of S6-phospho and normal Star-PAP proteins in HeLa cells after CKIα knockdown using S6-phospho and N-Term Star-PAP (normal Star-PAP) antibodies (Figure 4D). We observed localization of both S6-phospho and normal Star-PAP in the nuclear speckles (Figure 4D). Consistently, knockdown of CKIα (Figure 4C, Supplementary Figure S5B) resulted in the specific loss of S6-phospho Star-PAP in the nucleus with no effect on the localization of normal Star-PAP protein (Figure 4D). Moreover, in an *in vitro* kinase assay, FLAG- affinity purified Star-PAP was phosphorylated by CKIα but not by CKIIα (Supplementary Figure S3F). Consistently, CKIα but not CKIIα exhibited kinase activity toward a short Star-PAP N-terminal peptide (corresponding control peptide for S6-phospho used for raising anti S6-phospho antibody) (Supplementary Figure S3G and H). Together these results demonstrate that CKIα is the kinase that phosphorylates S6 residue on ZF Star-PAP.

Furthermore, we examined the effect of CKIα on the functional aspects of S6 phosphorylation. Knockdown of CKIα or treatment with CKI inhibitors CKI7 and IC261 reduced the expression of Star-PAP target HO-1 mRNA (Figure 4E). Knockdown of CKIIα did not have any effect on the expression of HO-1 mRNA levels. Similar results were observed with NQO-1 mRNA levels as well (data not shown) whereas Star-PAP non-target GCLC level was not affected by either knockdown or inhibition of CKIα (Figure 4F). To rule out the effect of other Star-PAP phosphorylations on the expression/3′-end processing of Star-PAP targets HO-1 and NQO-1 mRNA observed in this study, we treated HEK 293 cells with a large number of kinase inhibitors [SK1-I(sphingosine kinase), Tamoxifen (Ca^2+^-CAM, PKC), Staurosporin (PKC, several kinase), Rottlerin (PKCδ), SB203580 (P38-MAPK), Nimbolide (ERK), AG-490 (JAK), S-CR8(CDK) and CKI7] and analyzed the levels of HO-1 and NQO-1 mRNAs. None of the kinase inhibitors tested exhibits any effect on basal level mRNA expressions of HO-1 or NQO-1 except CKI7 (Supplementary Figure S4G). 3′-RACE assay also indicated specific effect of CKI7 on 3′-end processing of HO-1 mRNA (data not shown). However, there are reports of transcriptional regulation of HO-1/NQO-1 expression by several kinases (56–59). Together these results indicate that regulation of HO-1 and NQO-1 at the 3′-end required CKIα phosphorylation (S6) of Star-PAP.

To further assess the effect of loss of CKIα on Star-PAP interaction with PIPKIα and RNA binding, we carried out immunoprecipitation and RIP experiments respectively in HEK 293 cells after knockdown of CKIα (Figure 4H) or treatment with CKI7. Knockdown or inhibition of CKIα resulted in the loss of PIPKIα association with Star-PAP while control cells or DMSO treated cells showed normal association with PIPKIα (Figure 4G). RIP experiments using anti-FLAG antibody in HEK 293 cells stably expressing FLAG-Star-PAP^WT^ or -Star-PAP^S6A^ also demonstrated a similar loss of Star-PAP association with HO-1 UTR RNA on knockdown or inhibition of CKIα in the cell (data not shown). These results indicate that CKIα mediated phosphorylation of S6 Star-PAP modulates Star-PAP RNA binding and association with PIPKIα in the cell to control target mRNA expression.

### Star-PAP S6 phosphorylation occurs within the nucleus

To examine the role of S6 phosphorylation in the nuclear localization of Star-PAP, IF experiments were carried out using N-Term or S6-phospho antibody. While normal Star-PAP was localized at the nuclear speckles, there was detectable Star-PAP in the cytoplasm (Figure 5A) which increased upon transient expression of Star-PAP (Supplementary Figure S5C). S6-phospho Star-PAP was also seen in the nuclear speckles; however, endogenous S6-phospho Star-PAP detected in the cytoplasm was negligible (Figure 5A). To confirm the nuclear presence of S6-phospho Star-PAP, we analyzed the cytoplasmic and nuclear fractions in HEK 293 cells (indicated with lamin B and β-tubulin as nuclear and cytoplasmic markers after the fractionation) (Figure 5B). Star-PAP was immunoprecipitated from both the nuclear and cytoplasmic fractions and tested for the presence of S6-phospho Star-PAP. Immunoprecipitation showed the presence of Star-PAP in both nuclear and cytoplasmic fractions (Figure 5C). Consistent with the imaging data, a minor (negligible) portion of S6-phospho Star-PAP was detected in the immunoprecipitate of total Star-PAP from cytoplasmic fraction compared to that of nuclear fraction (Figure 5C). Western blot analysis on the nuclear and cytoplasmic fractions also demonstrated presence of normal Star-PAP both in cytoplasmic and nuclear fractions and S6-phospho Star-PAP predominantly in the nuclear fraction (Supplementary Figure S5D). Together these results demonstrate that S6-phospho Star-PAP is selectively retained inside the nucleus.

Knockdown of CKIα resulted in the loss of S6-phospho Star-PAP in the nucleus without changing the nuclear localization of normal Star-PAP. Similarly, when we inhibited CKIα in the cell with CKI7, there was no effect on normal Star-PAP nuclear localization, yet, there was no S6-phospho Star-PAP detected in the nucleus or in the cytoplasm (Figure 4D) indicating that there is no effect of S6 phosphorylation on the nuclear import of Star-PAP and that S6-phosphorylation likely occurs within the nucleus. To further explore this, we transiently expressed FLAG-Star-PAP^WT^ or -Star-PAP^S6A^ after knockdown of the endogenous Star-PAP in HeLa cells and carried out IF staining using anti-FLAG antibody or S6-phospho antibody. Interestingly, both Star-PAP^WT^ (Supplementary Figure S5C) as well as Star-PAP^S6A^ (Figure 5D) were detected equally in the nuclear speckles. There was no effect of the S6A mutation on Star-PAP nuclear localization as FLAG-Star-PAP^S6A^ exhibited normal localization in the nucleus (Figure 5D). However, there was no S6-phospho Star-PAP detected in the nucleus or in the cytoplasm in presence of the S6A mutation (Figure 5D). This was further confirmed using deletion of C-terminal end (encompassing the nuclear localization signal, NLS) on the FLAG-Star-PAP (Figure 5E) and control WT Star-PAP (Figure 5F). While Star-PAP^WT^ showed normal localization (Figure 5F, Supplementary Figure S5C), NLS deletion prevented nuclear localization of Star-PAP and all Star-PAPs were detected in the cytoplasm (Figure 5E). Interestingly, there was no S6-phospho Star-PAP detected in either the cytoplasm or in the nucleus (Figure 5E) demonstrating that S6 phosphorylation occurs inside the nucleus. Nuclear fractionation also demonstrated that while FLAG Star-PAP^WT^ was present in both nuclear and cytoplasmic fractions, S6A (phospho deficient) mutant or C-terminal (NLS) deletion (compromised for nuclear import) Star-PAP were neither detected in the nuclear nor in the cytoplasmic fractions (Figure 5G) reiterating that Star-PAP S6 phosphorylation occurs inside the nucleus. Concomitantly, CKIα that phosphorylates S6 on Star-PAP also localizes in the nuclear speckles (Supplementary Figure S5B) along with Star-PAP (16).

### S6 phosphorylation regulates select set of Star-PAP target messages

**Figure 6.**
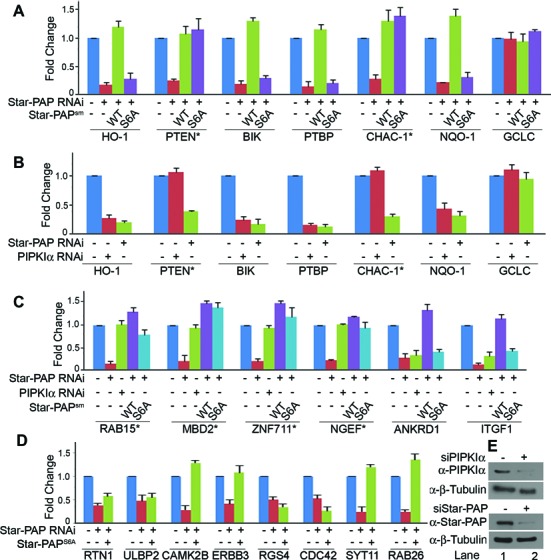
Star-PAP S6 phosphorylation together with PIPKIα determines specificity of target mRNA expression. qRT-PCR analysis of (**A**) Star-PAP target mRNAs as indicated with total RNA isolated from HEK 293 cells after Star-PAP knockdown rescued with stable expression of WT or S6A mutant Star-PAP; (**B**) after knockdown of PIPKIα or Star-PAP; (**C**) qRT-PCR as in (A) with select Star-PAP target mRNAs as indicated and (**D**) more Star-PAP target mRNAs as in (A) to screen for S6-phospho dependent target mRNAs. (**E**) Western blot analysis showing knockdowns of Star-PAP and PIPKIα. Wherever siRNA (−) is indicated in the figure, we have used control scrambled siRNA.

We have shown that CKIα mediated Star-PAP S6 phosphorylation and PIPKIα together regulates HO-1 expression and specific mRNA binding. To further study the role of S6 phosphorylation on the expression of Star-PAP target messages, we selected Star-PAP mRNA targets (HO-1, BIK, PTEN, PTBP2, CHAC1, NQO1 and the non-target control GCLC). Using qRT-PCR, we measured the mRNA levels encoded by these genes after knockdown of Star-PAP (Figure 6E) in HEK 293 cells in presence of stably expressed Star-PAP^WT^ and Star-PAP^S6A^. There was loss of mRNA expression of all the messages (HO-1, BIK, PTEN, NQO1, PTBP2 and CHAC1) on Star-PAP knockdown except the non-target GCLC (Figure 6A), all of which were rescued by stable expression of Star-PAP^WT^ in the cell (Figure 6A). However, S6A mutation rescued expression of some but not all messages studied. While CHAC1 and PTEN mRNA expressions were rescued, S6A mutation failed to rescue loss of HO-1, BIK, PTBP2 and NQO1 expressions (Figure 6A) indicating that S6 phosphorylation regulates select target mRNAs. This suggests a new mechanism of phosphorylation mediated target mRNA specificity.

### Star-PAP S6 phosphorylation along with PIPKIα determines Star-PAP target mRNA specificity

PIPKIα regulates Star-PAP through its interaction with Star-PAP in the ZF-region and controls expression of Star-PAP target messages. Interestingly, Star-PAP target CHAC1 mRNA expression was earlier reported to be independent of PIPKIα regulation (16). We have also shown that Star-PAP association with CHAC1 mRNA is independent of S6 phosphorylation and PIPKIα knockdown. To understand how many of the above messages were dependent on Star-PAP S6-phosphorylation and PIPKIα, we knocked down PIPKIα (Figure 6E) and examined the expression profiles of above mentioned genes. Interestingly, with the exception of PTEN and CHAC1 which were independent of S6-phosphorylation, PIPKIα knockdown resulted in the loss of all other messages (HO-1, BIK, NQO1 and PTBP2) (Figure 6B). Consistently, CHAC1 expression was independent of the knockdown or inhibition of CKIα as well (Supplementary Figure S5F). However, Star-PAP^WT^ or Star-PAP^S6A^ did not rescue the loss of expression of these transcripts due to PIPKIα knockdown (Supplementary Figure S5E). These results are consistent with our earlier microarray data (46) indicating that S6-phosphorylation and PIPKIα cooperates with each other to regulate a common set of target mRNAs.

To further explore this, several Star-PAP target mRNAs were selected from earlier microarray data (46) and examined the impact of S6A mutation on the mRNA levels. Two sets of mRNAs were selected, one (similar to HO-1) which was dependent on both Star-PAP and PIPKIα (CCNA1, MEGF10, CXCL2, ITFG1 and ANKDR1) and the other (similar to CHAC1) regulated only by Star-PAP but insensitive to PIPKIα knockdown (RAB15, NGEF, PPAP2C, ZNF711 and MBD2). As expected, qRT-PCR analysis revealed loss of expression of all mRNAs from both sets on Star-PAP knockdown (Figure 6C, Supplementary Figure S6A). However, PIPKIα knockdown resulted in a selective loss of only the first set of mRNAs with no effect on the expression of the second set of mRNAs (RAB15, NGEF, PPA2C, ZNF711 and MBD2) (Figure 6C; Supplementary Figure S6A). Interestingly, in a rescue experiment with stably expressed Star-PAP^WT^ or Star-PAP^S6A^, S6A mutant was unable to rescue the loss of expression specifically of the first set of mRNAs (which were sensitive to PIPKIα) after Star-PAP knockdown (Figure 6C). Also, the second set of mRNAs which were insensitive to PIPKIα knockdown were rescued equally by the S6A mutant and the WT Star-PAP (Figure 6C; Supplementary Figure S6A) indicating that second set of mRNA expression is insensitive to both S6-phosphorylation of Star-PAP and PIPKIα. Together, these results demonstrate that S6 phosphorylation is selective in controlling the expression of Star-PAP target mRNAs and that it functions in concert with PIPKIα. We further screened more S6-phospho dependent Star-PAP target mRNAs from our earlier microarray analysis of Star-PAP regulated genes (Figure 6D). A list of S6-phospho dependent and independent Star-PAP target mRNAs are shown in Supplementary Table S1.

## DISCUSSION

Star-PAP is nuclear PAP variant that regulates select mRNA targets in the cell (46). Star-PAP assembles distinct 3′-end processing complex and associates with unique proteins such as PIPKIα, CKIα/ϵ or PKCδ (2,60). Our results demonstrated that PIPKIα interaction with Star-PAP is dependent on S6 phosphorylation by serine kinase CKIα. However, PIPKIα and CKIα controlled only a specific subset of Star-PAP target mRNAs (46) mediated through the ZF domain at the Star-PAP N-terminus where PIPKIα binds. Concomitantly, S6 phosphorylation also regulates specific subset of Star-PAP target messages that are common with those of PIPKIα suggesting a signal-specific regulatory module of Star-PAP. For example, while apoptotic gene BIK is controlled by DNA damage and PKCδ (18), stress response proteins HO-1 and NQO-1 are controlled by oxidative stress and CKIα (16,17), mediated through S6 phosphorylation (this study) and phosphorylation at the catalytic domain (16,17).

**Figure 7.**
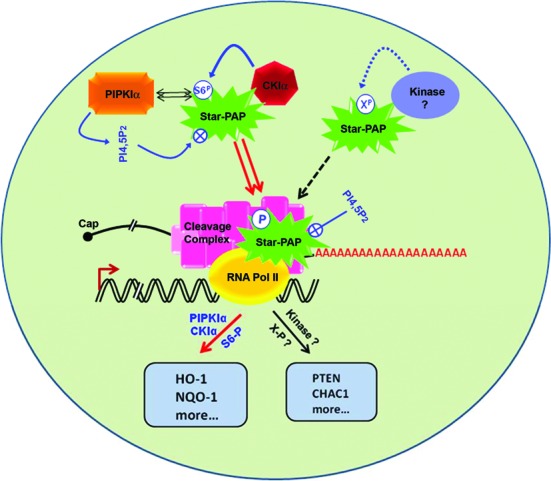
Model of phosphorylation mediated Star-PAP target mRNA specificity. Star-PAP S6 phosphorylation and PIPKIα that interacts via ZF-Star-PAP controls specific set of mRNAs including HO-1 and NQO-1. Other putative kinases with distinct signaling mediated specific target mRNAs are also depicted.

Phosphorylation of PAPs—both PAPα and Star-PAP regulate their activity (2). PAPα phosphorylation by kinases cdc2-cyclin B or ERK controls its activity (11–14,61,62). The new Star-PAP phosphorylation site identified in this study (S6) appears to regulate multiple aspects of Star-PAP function—from PIPKIα interaction to target mRNA specificity suggesting a key role of phosphorylation in overall regulation of Star-PAP function. A striking discovery of this study is that the S6 phosphorylation regulates only a select subset of mRNAs, which are also overlapping targets of PIPKIα. This indicates that PIPKIα and S6-phospho Star-PAP function together to regulate shared mRNA targets. The expressions of these messages were independent of other kinases including PKCδ that phosphorylates elsewhere on Star-PAP (18). This suggests a novel mechanism of Star-PAP target mRNA specificity determined by phosphorylation of Star-PAP (Figure 7). To our knowledge this is the first example of a PAP (or even a polymerase) where specificity of target gene expression is determined by phosphorylation status of the polymerase.

The identification of Star-PAP as a variant nuclear PAP indicated specificity of PAPs for mRNA selection, yet the mechanism of target mRNA recognition is not clear. Star-PAP binds a GC-rich sequence upstream of target poly(A) site (18,53) and putatively recognized a motif -AUA- *in vitro* (63) which likely provides specificity for Star-PAP mRNA targets. S6 phosphorylation could affect Star-PAP mRNA association either by its direct effect on the ZF motif or indirectly by altering the PIPKIα interaction that in turn regulates Star-PAP mRNA association. We have shown that S6 phosphorylation functions with PIPKIα and is independent of the Star-PAP ZF structure. Our results support the idea of phosphorylation induced mRNA specificity where Star-PAP targets regulated by S6 phosphorylation are independent of other putative kinases that phosphorylate Star-PAP (16). Therefore, different signaling pathways may induce phosphorylation of distinct residues on Star-PAP thus regulating specific transcripts (Figure 7).

While normal Star-PAP was detected both in the nucleus and the cytoplasm, S6 phosphorylation of Star-PAP was tightly associated within the nucleus. This suggests the possibility of Star-PAP shuttling between the cytoplasm and the nucleus wherein phosphorylation is required for nuclear retention (64). However, there is no direct evidence to show that Star-PAP shuttles between the nucleus and cytoplasm except that Star-PAP is present in both the cellular compartments that becomes primarily nuclear after S6 phosphorylation. Star-PAP S6 phosphorylation is not involved in its nuclear import but is primarily retained inside the nucleus. Our results suggest a likely role of S6 phosphorylation in Star-PAP nuclear retention, possibly by interaction with component such as PIPKIα. Clearly, not all Star-PAPs present in the nucleus are (S6) phosphorylated and the Star-PAP detected with the polyclonal antibody colocalized with S6-phospho Star-PAP (Supplementary Figure S6B). Nevertheless, S6-phospho Star-PAP was exclusively detected inside the nucleus.

Star-PAP associates with multiple kinases that includes PI4,5P_2_ sensitive kinase CKIα/ϵ and PKCδ in addition to yet unidentified kinases (16–18). Yet, the 3′-end processing of HO-1 and NQO-1 are specific only to CKIα. Moreover, the exact phosphorylation sites of any of the kinases were unknown. This study for the first time identified an *in vivo* phosphorylation site of CKIα on Star-PAP. Interestingly, CKIα mediated phosphorylation itself is stimulated by stress (17,65). However, the stress stimulation appears to be specific to target sites or proteins and not a general activation of CKI activity. Although CKIα substrates such as Star-PAP are stimulated by oxidative stress, there are several CKIα target proteins that are not regulated by oxidative stress. Even in the case of Star-PAP, the new CKIα site (S6) identified in this study is insensitive to oxidative stress stimulation of the cell. Yet, CKIα mediated phosphorylation in the Star-PAP PRR is stimulated by oxidative stress (17) indicating a signal specific stimulation of CKIα kinase activity. This indicates an alternative regulation of CKIα activity where oxidative stress stimulation of Star-PAP phosphorylation is determined by distinct CKIα site/motif to be phosphorylated. However, what actual CKIα phosphorylation motif(s) (or sites) is a target of oxidative stress stimulation is still unclear. Our results suggest that CKIα mediated S6 phosphorylation of Star-PAP is required for basal activity and specificity toward mRNAs, but that additional phosphorylation events are required for Star-PAP activity downstream of signals such as oxidative stress.

## Supplementary Material

SUPPLEMENTARY DATA
