# Supplementary material for: Phosphorylation regulates the Star-PAP-PIPKIα interaction and directs specificity toward mRNA targets
Source: Nucleic Acids Res. 2015 Jul 2;43(14):7005–20. doi: 10.1093/nar/gkv676 (PMC4538844; doi:10.1093/nar/gkv676)
Supplement: SUPPLEMENTARY DATA [file supp_43_14_7005__index.html]

Phosphorylation regulates the Star-PAP-PIPKIα interaction and directs specificity toward mRNA targets — Phosphorylation regulates the Star-PAP-PIPKIα interaction and directs specificity toward mRNA targets — SUPPLEMENTARY DATA 

# Phosphorylation regulates the Star-PAP-PIPKIα interaction and directs specificity toward mRNA targets

## SUPPLEMENTARY DATA

- SUPPLEMENTARY DATA
