# Supplementary material for: Phosphorylation regulates the Star-PAP-PIPKIα interaction and directs specificity toward mRNA targets
Source: Nucleic Acids Res. 2015 Jul 2;43(14):7005–20. doi: 10.1093/nar/gkv676 (PMC4538844; doi:10.1093/nar/gkv676)
Supplement: SUPPLEMENTARY DATA [file supp_gkv676_nar-00240-a-2015-File009.pdf]

Supplementary Data

Title

Phosphorylation regulates the Star-PAP-PIPKIα interaction and directs specificity toward mRNA targets

Nimmy Mohan[1*], Sudheesh AP[1*], Nimmy Francis[1], Richard Anderson[2], and Rakesh S. Laishram[1#]

[1] *Cancer Research Program, Rajiv Gandhi Centre for Biotechnology, Trivandrum 695014, India*

[2] *School of Medicine and Public Health, University of Wisconsin-Madison 53706, USA*

[*] *These authors contributed equally to this work*

[#] *Corresponding Author*

Running Title: Phosphorylation mediated Star-PAP mRNA target specificity

[#]Corresponding Address:

Rakesh S. Laishram

Rajiv Gandhi Centre for Biotechnology

Thycaud Post, Poojappura

Trivandrum – 695014, India

E mail: laishram@rgcb.res.in

Phone: +91-0471-2529592

List of Antibodies:

Rabbit polyclonal anti-CKIα (Bethyl lab), Rabbit polyclonal anti-CKIε (Bethyl lab), Rabbit polyclonal anti-CKIIα (Bethyl lab), Rabbit monoclonal Lamin B2 (Abcam), Rabbit monoclonal anti beta Tubulin (Novus), Mouse monoclonal anti alpha Actin (Novus), Mouse anti-His tag monoclonal antibody (Cell Biolabs), Mouse anti-GST tag monoclonal antibody (Cell Biolabs), Mouse monoclonal anti-FLAG M2 (Sigma), Rabbit monoclonal anti-FLAG (Sigma), Mouse monoclonal anti-PIPK1α (Santa Cruz), Rabbit polyclonal anti-PIPKIα (Mellman et al, 2008), Mouse monoclonal anti SC-35 (Abcam), Rabbit polyclonal anti-RNA polymerase II (Bethyl lab), Rabbit polyclonal anti-CPSF 160 (Bethyl Lab), Rabbit polyclonal Heme Oxygenase 1 (Novus), Rabbit polyclonal anti-Star-PAP (Mellman et al, 2008), Rabbit polyclonal anti-N-Term Star-PAP (Mellman et. al, 2008), Rabbit polyclonal anti-S6 phopsho Star-PAP (this study), Rabbit polyclonal anti-N-Term non-phopsho Star-PAP (this study) were used for Western blot, IP or RIP experiments.

Gene specific forward primers used for 3'-RACE:

1. HO-1 5'- CTTACACTCAGCTTTCTGGTG -3'
2. NQO1 5'- ACTGATGACTTACTATGGGA - 3'
3. GAPDH 5'-TTTGGCTACAGCAACAGGGT- 3'
4. GCLC 5'-GCATTATTGACGAACTGGCTACA-3'

Primers used for 3'-end cleavage:

1. HO-1 Clv FP: 5'-GGCACTGTGGCCTTGGTCTAA-3'
   Clv RP: 5'-TCCTACCGAGCACGCAAGAA-3'

2. NQO-1 Clv FP: 5'- TGCCTTCATCTTCACTGCAA-3'
   Clv RP: 5'- TTGTCAAGCCAGTCACCAAG -3'

3. GCLC Clv FP: 5'-ATGCCTGGTTTTCGTTTGCA-3'
   Clv RP: 5'-AGCTGTGGAACTCACACACACTCA-3'

4. GAPDH Clv FP: 5'-CACACTGAATCTCCCCTCCT-3'
Clv RP: 5'- TTGACACAAGCCCAGCTTC-3'

5. CHAC1 Clv FP: 5'-TGTTTTCTTGTCCCTCTGTCTG-3'
Clv RP: 5'- CAAACCCCCATCTGAGAAAG-3'

Primers for RIP analysis:

1. HO-1 FP: 5'-GGCACTGTGGCCTTGGTCTAA-3'
RP: 5'- TCCTACCGAGCACGCAAGAA -3'

2. NQO-1 FP: 5'- TGCTCTCGACAGTATCCACAA-3'
RP: 5'-AGTTGTCAAGCCAGTCACCAA -3'

3. GAPDH FP: 5'-CACACTGAATCTCCCCTCCT-3'
RP: 5'- TTGACACAAGCCCAGCTTC-3'

4. CHAC1 FP: 5'-TGTTTTCTTGTCCCTCTGTCTG-3'
RP: 5'- CAAACCCCCATCTGAGAAAG-3'

Primers for quantitative RIP:

1. HO-1 FP: 5'-GGCACTGTGGCCTTGGTCTAA-3'
RP: 5'-TCCTACCGAGCACGCAAGAA-3'

2. NQO-1 FP: 5'- TGCCTTCATCTTCACTGCAA-3'
RP: 5'- TTGTCAAGCCAGTCACCAAG-3'

3. GAPDH FP: 5'-CACACTGAATCTCCCCTCCT-3'
RP: 5'- TTGACACAAGCCCAGCTTC-3'

4. CHAC1 FP: 5'-TGTTTTCTTGTCCCTCTGTCTG-3'
RP: 5'- CAAACCCCCATCTGAGAAAG-3'

Primers for splicing assay:

1. HO-1 FP: 5’-AACTTTCAGAAGGGCCAGGT-3’
RP: 5’-CTCCTTGTTGCGCTCAATCT-3’

2. NQO-1 FP: 5’- GTCATTCTCTGGCCAATTCA-3’
RP: 5’- GCTGCTTGGAGCAAAATACA-3’

3. GCLC FP: 5’- AAAAGTCCGGTTGGTCCTGT-3’
RP: 5’- CTCTGGTCTCCAAAGGGTAGG-3’

4. GAPDH FP: 5’- TTCACCACCATGGAGAAGG-3’
RP: 5’- TTCACACCCATGACGAACA-3’

Primers used in quantitative real time PCR:

1. HO-1 FP: 5'- CCACCAAGTTCAAGCAGCTCTA - 3'
RP: 5'- GCTCCTGCAACTCCTCAAAGAG -3'

2. BIK FP: 5'-TCTTGATGGAGACCCTCCTG-3'
RP: 5'-GTCCTCCATAGGGTCCAGGT-3'

3. GCLC FP: 5'- AAGTTCTTGAAACTCTGCAAGAGAAGG -3'
RP: 5'- GCCTCAACTGTATTGAACTCGGAC -3'

4. GAPDH FP: 5'- GAAGGTCGGAGTC AACGGATTT -3'
RP: 5'- GAATTTGCCATGGGTGGAAT -3'

5. PTBP2 FP: 5'-ACAGTTAGCGAGAGTGCAGTGA-3'
RP: 5'-GGAGCAAAGCTTGAAACTGG-3'

6. PTEN FP: 5'- AAGCTGGAAAGGGACGAACT-3'
RP: 5'-ACACATAGCGCCTCTGACTG-3'

7. CHAC1 FP: 5'-GTGACGCTCCTTGAAGATCA-3'
RP: 5'-CACATAGGCCAATGCCTTC-3'

8. NQO1 FP: 5'- GAACTTCAATCCCATCATTTCCAG -3'
RP: 5'- CAGCTTCTTTTGTTCAGCCACAAT -3'

9. RAB15 FP: 5'-CATCATGAAGTGGGTCAGTG-3'
RP: 5'-TGCGTACCAGAGGTCTTCAT-3'

10. MBD2 FP: 5'-AGGCTACAAGGACTTAGTGCATC-3'
RP: 5'-AGCACTGGCAACAGCAGATA-3'

11. ZNF711 FP: 5'- ATTCAAGCAGCTGGAGGTGT-3'
RP: 5'- TGTGAACCATCACTGCCAAT-3'

12. NGEF FP: 5'-TCACCTACGTCAGCAATCAG-3'
RP: 5'-TCTGGAAAGGCAGGATGA-3'

13. PPAP2C FP: 5'-TCAACTGCTCGGTCTATGTG-3'
RP: 5'-ATGCAGTACATCCCAAAGGA-3'

14. ANKRD1 FP: 5'-TGGATGTGCCTACGTTTCTG-3'
RP: 5'-GGCTCCAGCTTCCATTAACTT-3'

15. CXCL2 FP: 5'-GCAGGGAATTCACCTCAAGA-3'
RP: 5'-GACAAGCTTTCTGCCCATTC-3'

16. ITFG1 FP: 5'- CTTTGTGCCATTTGTGGATG-3'
RP: 5'- GCCTCTTCACAGCTTGCATTA-3'

17. CCNA1 FP: 5'- AAACTGCAGCTCGTAGGAACA-3'
RP: 5'-AGAAACTGGTTGGTGGTTGG -3'

18. MEGF10 FP: 5'-TAAAAATGGGGCTTCATGCT-3'
RP: 5'- CATAAAAACCAGGGGAGCAG-3'

19. RGS4 FP: 5’-AAGTACGCTCAAAGCCGAAG-3’
RP: 5’-GTTGTGGGAAGAATTGTGTTCA-3’

20. CDC42 FP: 5’-TACGACCGCTGAGTTATCCA-3’
RP: 5’-TTGAGTCCCAACAAGCAAGA-3’

21. SYT11 FP: 5’- TGACCAGGGACATCATCAAA-3’
RP: 5’- ACCGGTGATATCCATCTTCG-3’

22. RTN1 FP: 5’- CATCGTGTTTGGGAGTTTCC-3’
RP: 5’- AAGGGTGATCTCAAGCTCCA-3’

23. ULBP2 FP: 5’- TACAGAGCAACTGCGTGACA-3’
RP: 5’- TTTCTGGCTCCAGGATGAA-3’

24. RAB26 FP: 5’-ATTCAAGGATGGTGCTTTCC-3’
RP: 5’-CACATCTGCAGCTTCACCTT-3’

25. ZCCHC12 FP: 5’-TGTGCAGCTGATTGCAGA-3’
RP: 5’-AAGAGCGCGTCTTTATTTCAC-3’

26. TCEA2 FP: 5’- AACCGTGTACGGAGTCGTATCT-3’
RP: 5’- TACGGATCTCCTTCAGCTCATC-3’

27. ASCC3 FP: 5’- AGCCTTTGCCTTCCCAATAC-3’
RP: 5’- TTGGTAAAGGCTGAAGATCCA-3’

28. CAMK2B FP: 5’- TCCTGAAGCCAAAAACCTCA-3’
RP: 5’- ATCATGGATGCTACCGTGGA-3’

29. ERBB3 FP: 5’-TTGAACTGGACCAAGGTGCT-3’

RP: 5’-TTTCGACAGGACAAGCACTG-3’

30. Star PAP (cds) FP: 5’-GTCCATGGCTGTGATCTTGA-3’
RP: 5’-TTCAAAGTCCAGGGCTTCAG-3’

31. Star PAP (UTR) FP: 5’-ACAGTCTCCCGTTTCCTTTC-3’
RP: 5’-AGCTGTGCGAACTGTAGGAA-3’

32. Flag NQO1 FP: 5’-ATGGATTACAAGGATGACGACG-3’
RP: 5’- CCTCCTTCATGGCATAGTTGAA-3’

siRNA Oligos used:

1. Control scrambled non-targeting: AGGUAGUGUAAUCGCCUUG
2. Star-PAP: GUGUGUUUGUCAGUGGCUU
2. PIPKIα: GAAGUUGGAGCACUCUUGG
3. CKIα: On target Smart pool (Dharmacon)
4. CKIIα: On target Smart pool (Dharmacon)
5. CKIε: On target Smart pool (Dharmacon)

Supplementary Figure Legend:

Supplementary Figure 1: S6 Star-PAP phosphorylation and its role in target mRNA processing. (A) FLAG affinity purified Star-PAP complex and Star-PAP primary sequence indicating Serine 6 phosphorylation site and peptides detected in the mass spectrometry analysis. (B) Predicted structure of Star-PAP ZF-region (1-50 amino acids) using ITASSER protein prediction program. (C-D) 3'-RACE assay of NQO-1 (C) and Star-PAP non-target GAPDH (D) as described in Fig. 1E. (E) Fold reduction of HO-1 expression after Star-PAP knockdown in presence of stable expressions of WT or S6A mutant Star-PAP in HEK 293 cells.

Supplementary Figure 2: Star-PAP knockdown or exogenous expression of mutant S6A Star-PAP is splicing independent. (A) Quantification of relative levels of endogenous Star-PAP and exogenously expressed Star-PAP^{WT} and Star-PAP^{S6A} relative to internal control β-Tubulin as

shown in Fig. 1D. (B) qRT-PCR of Star-PAP mRNA expression using primer pairs in the Star-PAP reading frame (cds) or at the UTR (absent in the exogenous Star-PAP^{sm} construct used for transfection) under the similar conditions described in Fig 1D. Star-PAP^{S6A} or Star-PAP^{WT} indicated is after siRNA knockdown of Star-PAP. (C) Schematic of reporter mini gene construct where FLAG-NQO-1 cds (without the introns) is expressed from pCMV promoter and is driven by either NQO-1 UTR or control SV40 UTR (hence splicing independent). (D) Western blot analysis of FLAG-NQO-1 using anti -FLAG antibody after transient expression of reporter construct from (C) in HEK 293 cells after Star-PAP knockdown. (E) qRT-PCR analysis of FLAG-NQO-1 reporter expression using a forward primer at the FLAG sequence and reverse primer from NQO-1 reading frame after transient expression of NQO-1 UTR driven or SV40 driven reporter constructs in HEK 293 cells as indicated. (F) 3'-RACE assay of NQO-1 or control GAPDH using the constructs and conditions as describe above. (G) Schematic of analysis of splicing efficiency and uncleaved pre-mRNA. A pair of primer across the cleavage site is used to measure the uncleaved pre-mRNA, and splicing efficiency by using a pair of primer across two exons as indicated. Both splicing and cleavage efficiencies are expressed relative to the total mRNA measured using primers within the coding region as indicated. (H-J) qRT-PCR to measure the uncleaved pre-mRNA (I), splicing efficiency (H) and total mRNA (J) levels of Star-PAP target HO-1 mRNA and control GCLC mRNA from HEK 293 cells after Star-PAP knockdown or in presence of exogenous expression of WT and S6A mutant after Star-PAP knockdown. Cleavage and splicing efficiencies are expressed relative to the total mRNA and presented as fold changes with respect to mRNA levels from control HEK 293 cells. Star-PAP^{S6A} or Star-PAP^{WT} indicated is after siRNA knockdown of Star-PAP.

Supplementary Figure 3: Characterisation of S6-phospho Star-PAP antibody. (A) Western blot analysis of normal and S6-phospho Star-PAP after Star-PAP knockdown in HEK 293 cells. (B) Peptide competitions in Western blot of S6-phopsho Star-PAP in presence of peptides as indicated. (C) Peptide competitions as in B of N-term Star-PAP antibody. (D) IF of S6-phopsho and N-Term Star-PAP in presence of control or Star-PAP knockdown in HeLa cells. (E) Peptide competitions of S6-phopsho Star-PAP in IF experiment as indicated. (F) In vitro kinase assay using FLAG-Star-PAP (heat inactivated) as substrate to test the kinase activities of CKIα and CKIIα. Silver stained Star-PAP is shown below. (G) In vitro kinase assay as in (F) but using short N-terminal Star-PAP peptide (14 amino acid), corresponding non-phospho peptide described in (B). Silver stained gel of the peptide is indicated below. (H) In vitro kinase assay as in (F) but using casein a substrate. Both casein and FLAG Star-PAP were analysed on 10% SDS PAGE, and N-Term peptide was analysed on a 25% SDS PAGE.

Supplementary Figure 4: (A) Immunofluorescence experiment using S6 Phospho and SC35 antibodies in HeLa cells in presence and absence of tBHQ stimulation of the cell. (B) qRIP analysis of Star-PAP and RNA Pol II association with HO-1 UTR RNA under conditions as indicated. (C-F) Immunoprecipitation of FLAG-Star-PAP from HEK 293 cells with stable expressed Flag-Star-PAP^{WT}, -Star-PAP^{S6A} and transiently expressed Star-PAP$^{\Delta PRR}$, Star-PAP^{C18S,C21S} to analyse associated proteins as indicated. (G) qRT-PCR analysis to measure total mRNA levels of Star-PAP target HO-1 and NQO-1 from total mRNA isolated form HEK 293 cells after treatment with various kinase inhibitors at concentrations [SK1-I, (2.5 µM), Tamoxifen (20 µM), Staurosporin (50 nM), Rottlerin (2 µM), SB203580 (3 µM), Nimbolide (1.25 µM), AG-490 (20 µM), S-CR8 (2 µM).

Supplementary Figure 5: (A) Star-PAP schematic domain architecture of ZF motif showing S6 phosphorylated region and the two Cys residues in the core ZF structure. (B) IF experiment of CKIα in presence of control or CKIα knockdown. (C) IF of S6-phopsho and N-Term Star-PAP in presence of FLAG-Star-PAP^{WT} expressed in HeLa cells. (D) Nuclear and cytoplasmic fractions showing S6-phospho and normal Star-PAP as indicated. (E) qRT-PCR of Star-PAP target mRNAs in presence of stable expressed WT or S6A Star-PAP after PIPKIα knockdown as indicated. (F) qRT-PCR of CHAC1 expression profile after knockdowns of Star-PAP, PIPKIα, CKIα or inhibitor CKI7 treatment, and in presence of exogenously expressed Star-PAP^{WT} and Star-PAP^{S6A}.

Supplementary Figure 6: (A) qRT PCR experiments as in Fig. 6C for mRNAs as indicated. (B) IF experiment S6-phopsho and N-Term (normal) Star-PAP co-localisation in HeLa cell. N-Term Star-PAP antibody was conjugated with Cy3 fluorescent dye (Abcam) to study the co-localisation.

Supplementary Figure 1

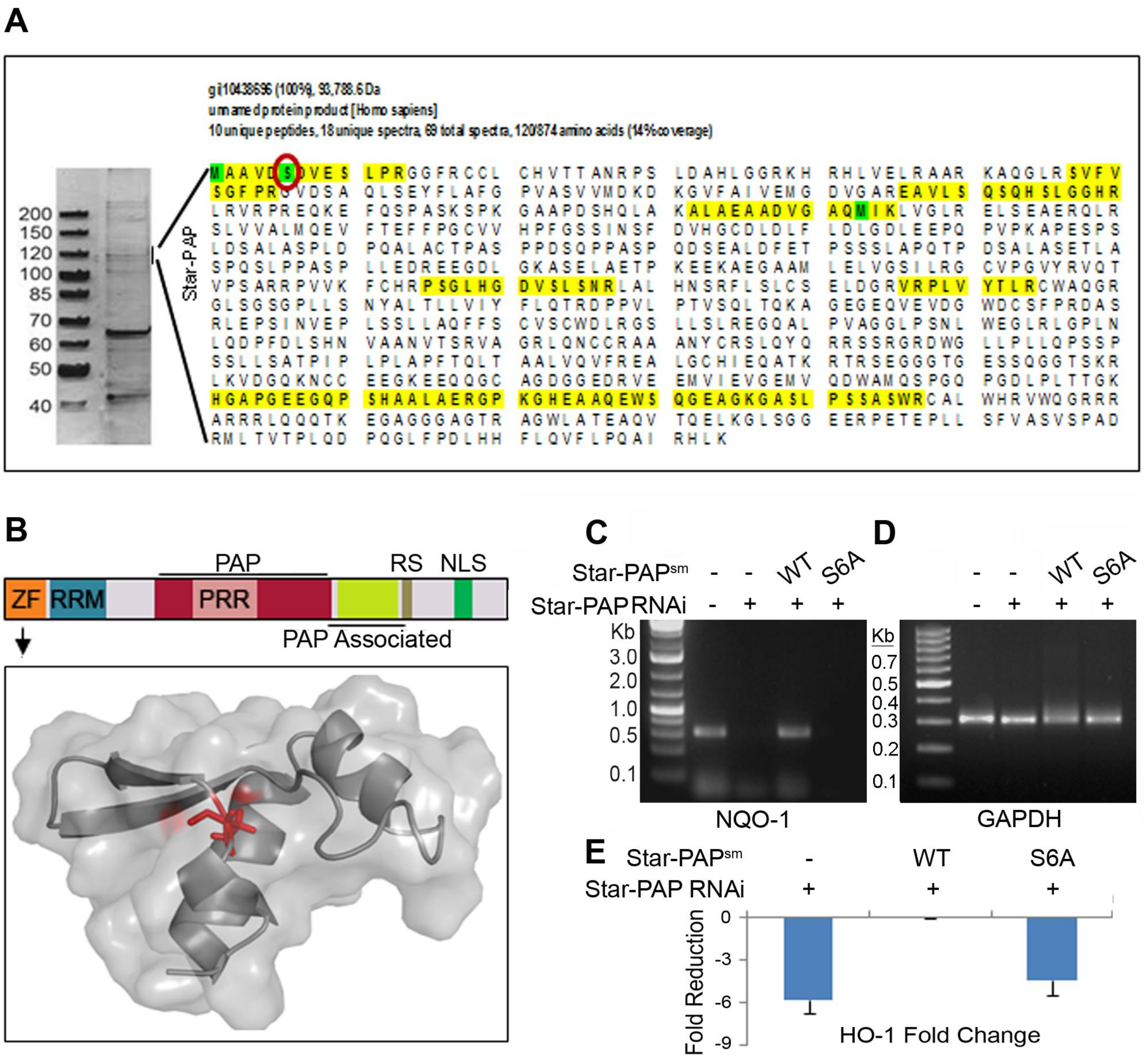

Supplementary Figure 2

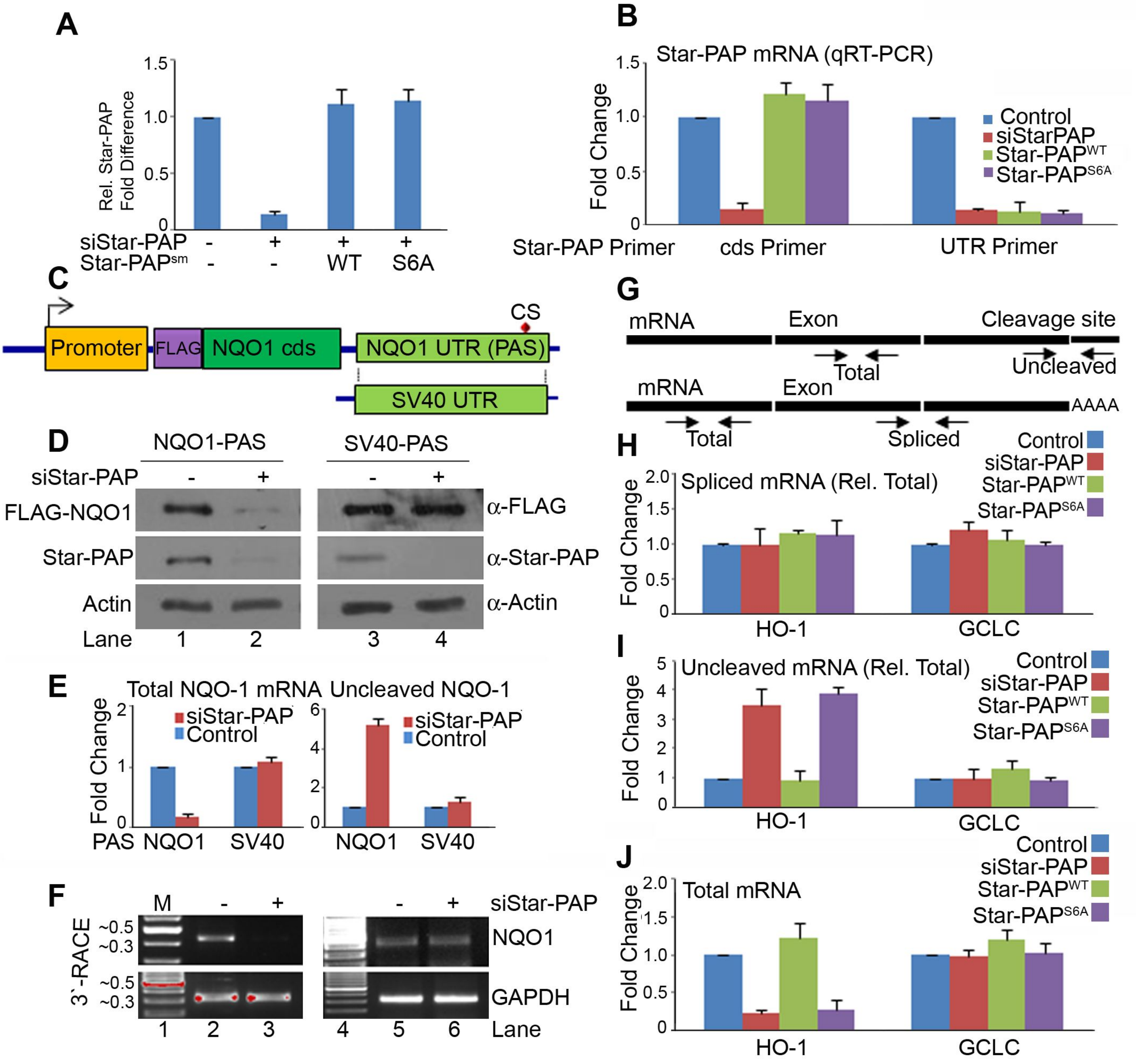

Supplementary Figure 3

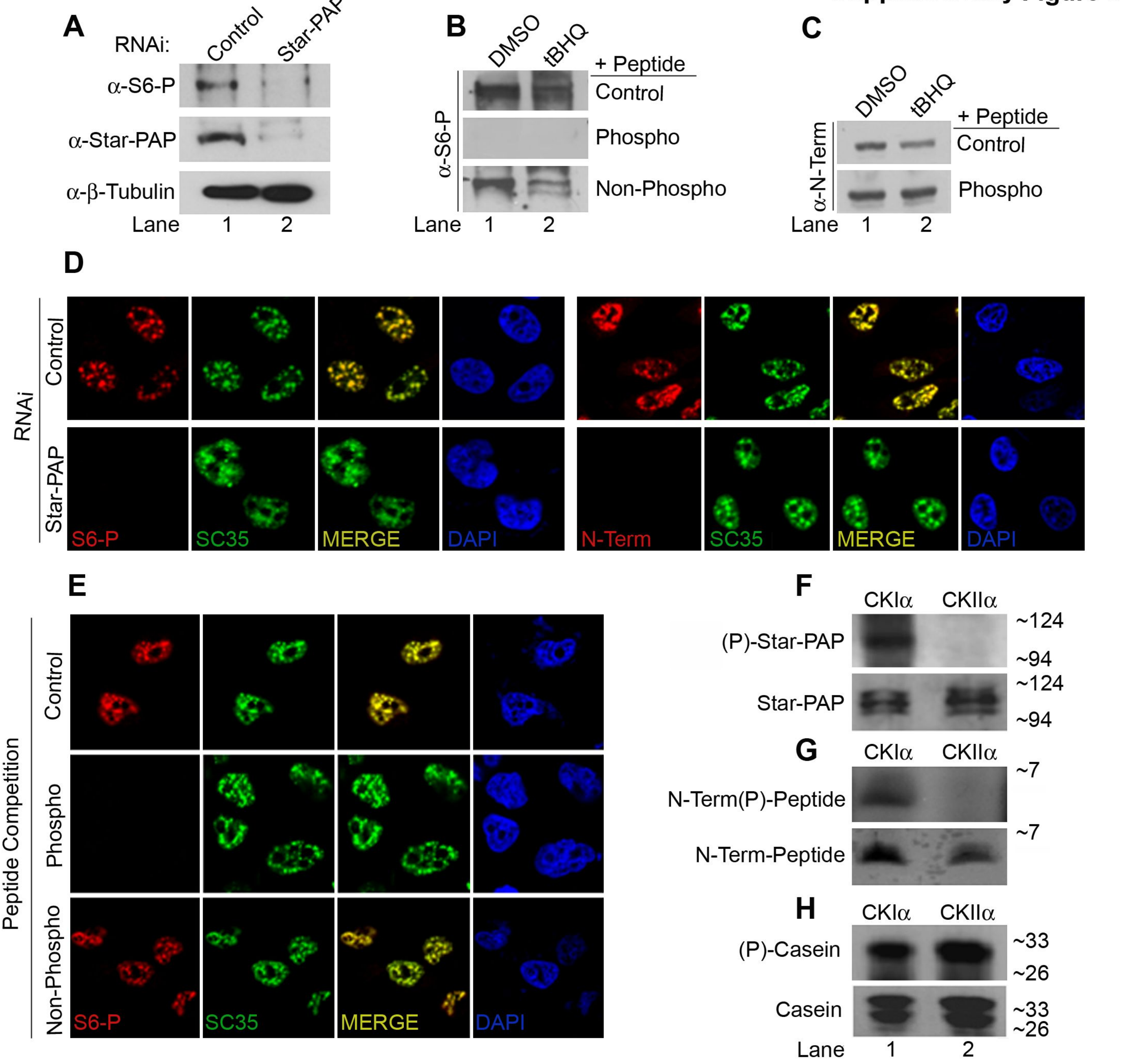

Supplementary Figure 4

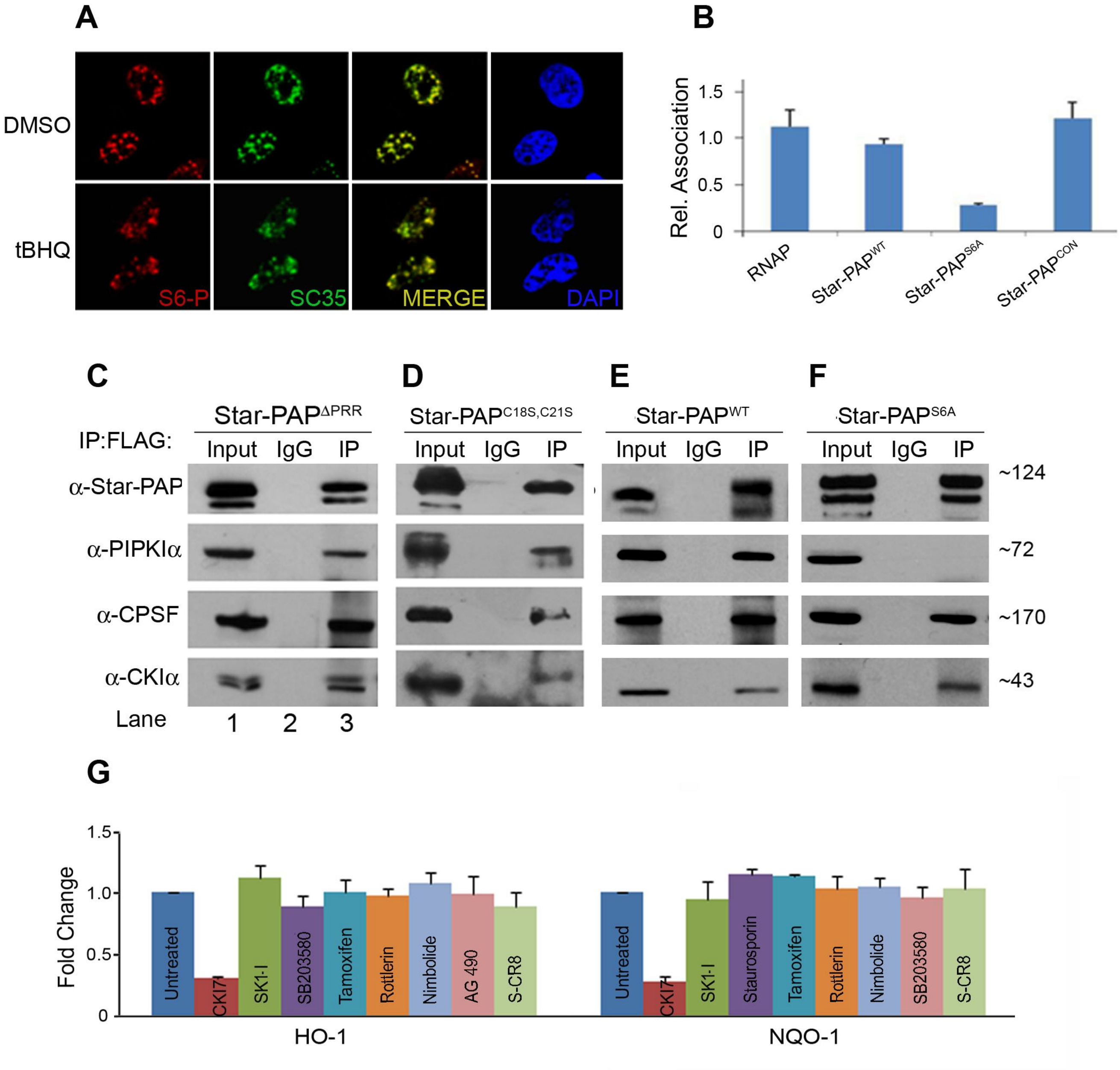

Supplementary Figure 5

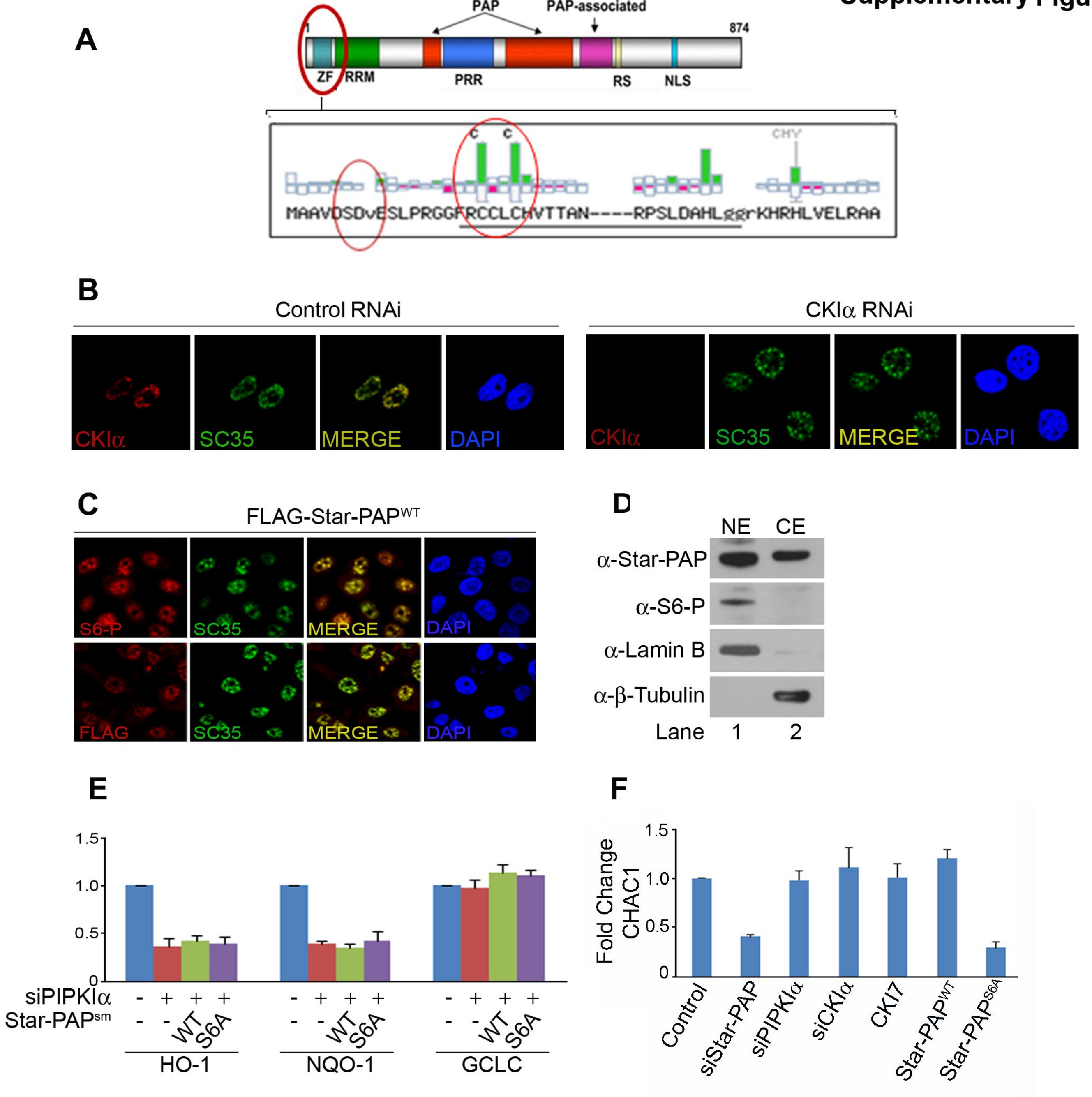

Supplementary Figure 6

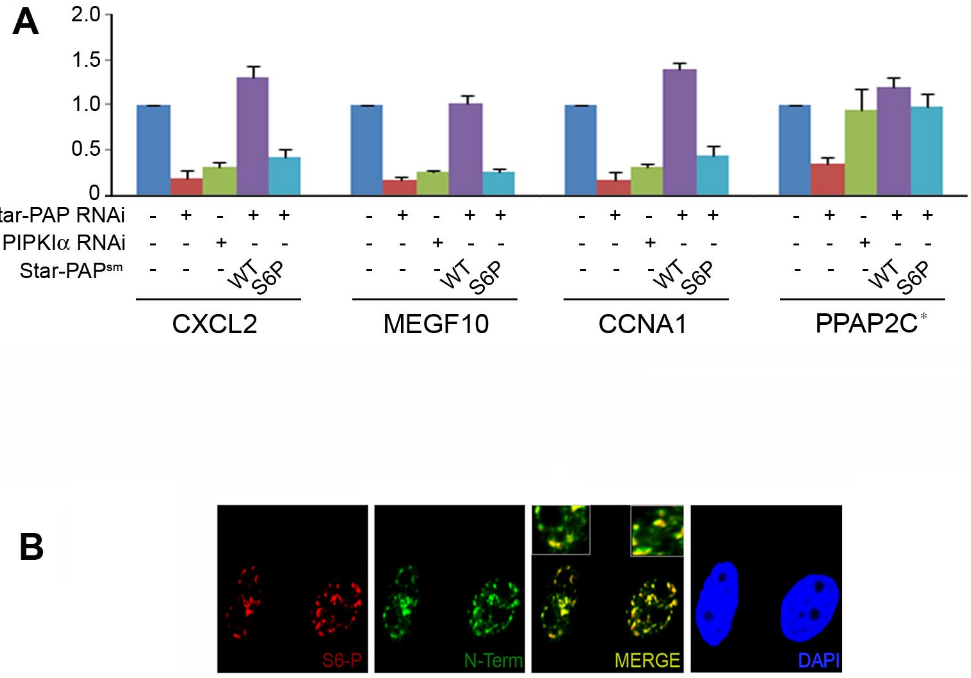

Supplementary Table 1: Selected list of S6-phospho dependent and independent Star-PAP target mRNAs

**S6-phospho dependent targets**	**S6-phospho independent targets**
HO-1 (*Heme oxygenase 1*)	PTEN (*Phosphatase and tensin homolog*)
BIK (*BCL2-interacting killer*)	CHAC1 (*Cation transporter regulator like 1*)
PTBP2 (*Polypyrimidine tract binding protein 2*)	RAB26 (*RAB26, member RAS oncogene family*)
NQO1 (*NAD(P)H quinone oxidoreductase 1*)	*ZCCHC12 (Zinc finger, CCHC domain containing 12*)
ANKRD1 (*Ankyrin repeat domain 1, cardiac muscle*)	ASCC3 *(Activating signal co integrator 1 complex subunit 3*)
ITGF1 (*Integrin alpha FG-GAP repeat containing 1*)	CAMK2B (*Calcium/calmodulin-dependent protein kinase II beta*)
CXCL2 (*Chemokine (C-X-C motif) ligand 2*)	ERBB3 (*erb-b2 receptor tyrosine kinase 3*)
MEGF10 (*Multiple EGF-like-domains 10*)	ZNF711 (*Zinc finger protein 711*)
CCNA1 (*Cyclin A1*)	NGEF (*Neuronal guanine nucleotide exchange factor*)
RGS4 (*Regulator of G-protein signalling 4*)	MBD2 (*Methyl-CpG binding domain protein 2*)
CDC42 (*Cell division cycle 42*)	PPAP2C (*Phosphatidic acid phosphatase type 2C*)
SYT11 (*Synaptotagmin XI*)	RAB15 (RAB15, *member RAS oncogene family*)
RTN1 (*Reticulon 1*)	LETM2(*Leucine zipper-EF-hand containing transmembrane protein 2*)
ULBP2 (*UL16 binding protein 2*)	MCAM(*Melanoma cell adhesion molecule*)
TCEA2 (*Transcription elongation factor A (SII), 2*)	GRK5(*G protein-coupled receptor kinase 5*)
